# Continuous Formation of Limonene Carbonates in Supercritical Carbon Dioxide

**DOI:** 10.1021/acs.oprd.2c00143

**Published:** 2022-09-16

**Authors:** Philipp Mikšovsky, Elias N. Horn, Shaghayegh Naghdi, Dominik Eder, Michael Schnürch, Katharina Bica-Schröder

**Affiliations:** † Institute of Applied Synthetic Chemistry (E163), TU Wien, Getreidemarkt 9/E163, 1060 Vienna, Austria; ‡ Institute of Materials Chemistry (E165), TU Wien, Getreidemarkt 9/E165, 1060 Vienna, Austria

**Keywords:** continuous flow chemistry, supercritical carbon dioxide, supported ionic liquid phase, bioderived cyclic carbonates, tetrabutylammonium halide, ethyl methyl imidazolium halide

## Abstract

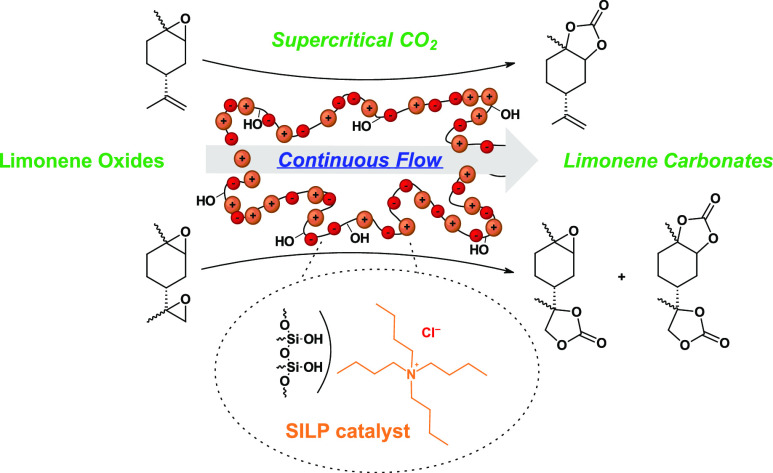

We present a continuous flow method for the conversion of bioderived limonene oxide and limonene dioxide to limonene carbonates using carbon dioxide in its supercritical state as a reagent and sole solvent. Various ammonium- and imidazolium-based ionic liquids were initially investigated in batch mode. For applying the best-performing and selective catalyst tetrabutylammonium chloride in continuous flow, the ionic liquid was physisorbed on mesoporous silica. In addition to the analysis of surface area and pore size distribution of the best-performing supported ionic liquid phase (SILP) catalysts via nitrogen physisorption, SILPs were characterized by diffuse reflectance infrared Fourier transform spectroscopy and thermogravimetric analysis and served as heterogeneous catalysts in continuous flow. Initially, the continuous flow conversion was optimized in short-term experiments resulting in the desired constant product outputs. Under these conditions, the long-term behavior of the SILP system was studied for a period of 48 h; no leaching of catalyst from the supporting material was observed in the case of limonene oxide and resulted in a yield of 16%. For limonene dioxide, just traces of leached catalysts were detected after reducing the catalyst loading from 30 to 15 wt %, thus enabling a constant product output in 17% yield over time.

## Introduction

The use of bioderived chemicals has attracted increasing attention in the past years in order to reduce the dependence on crude oil as limited feed stock.^1,2^ In this context, cyclic carbonates with an increasing annual production provoked by applications as electrolytes in lithium ion batteries as well as aprotic polar solvents or monomeric building blocks for polyurethanes are compounds of scientific as well as industrial interest.^3,4^ Suitable renewable starting materials for cyclic carbonates are oils and fatty acid,^5−9^ terpenes^10^ like limonene and carvone, and furfural derivatives.^11^ Additionally, limonene is a feedstock of high potential displayed in a global market of approximately 314 million US$ in 2020 and a global annual production of 43 Mt of limonene.^12^

One of the most important synthetic strategies for the synthesis of cyclic carbonates is the catalytic coupling of epoxides with carbon dioxide (CO_2_).^13^ CO_2_ is a widely and commonly used raw material of high abundance. Considering CO_2_ as a greenhouse gas, it is additionally of general interest to develop techniques for CO_2_ valorization. CO_2_ not only is nowadays used as a C1 building block for the synthesis of bulk chemicals like methanol or formic acid but is also increasingly used for the production of higher value chemicals. However, apart from the advantageous properties of being non-flammable and non-toxic, the high stability and therefore low reactivity of CO_2_ pose a challenge for the development of suitable catalytic systems. This challenge was accepted by the scientific community as well as the industry being reflected in reviews of the past years.^14−17^

In general, for the production of cyclic carbonates, derived from CO_2_ and epoxide, various catalytic systems on inorganic bases like metal complexes, metal oxides, and alkali metal halides as well as organic catalysts like organic bases, hydrogen donors, or ionic liquids were investigated in the past years.^18−21^

For cyclic limonene carbonates, various metal catalysts based on aluminum,^22,23^ lanthanum,^24^ iron,^25^ cobalt,^26^ scandium and yttrium,^27^ and calcium^6,28^ were studied. Additionally, examples with tungstate ionic liquids^29^ and ionic liquids^30−35^ are published. In various publications, ionic liquids were used as co-catalysts.^23−26,36,37^ However, only tetrabutyl ammonium halide-based ionic liquids were studied as single catalysts for the production of limonene mono- and biscarbonates.

In combination with supercritical CO_2_ (scCO_2_, *T*_c_: 31.0 °C, *p*_c_: 7.38 MPa),^38^ ionic liquids show a particular property of high value. The high solubility of scCO_2_ in ionic liquids^39^ makes ionic liquids ideal candidates as reaction media in combination with scCO_2_ for catalytic processes being favorable over the use of stochiometric amounts of reactants with regard to considerations of sustainability.^40,41^ In contrast, ionic liquids show extremely low solubility in scCO_2_, thus rendering them attractive for immobilized catalytic phases in heterogeneous catalysis,^42−44^ where leaching of the catalyst from the supporting material can be an issue.^45^ Further studies on the solubility of scCO_2_ in ionic liquids and *vice versa* are summarized in the stated publications.^46,47^

For the immobilization of ionic liquids toward continuous flow processes, supported ionic liquid phases (SILPs) are a well-known and widely used concept for catalytic and numerous other applications.^48,49^ An SILP material contains a thin film of ionic liquid on the supporting material, e.g., mesoporous silica, which offers a high surface area that is advantageous for catalytic processes and is able to overcome mass transfer limitations due to short diffusion lengths in the thin film.^50^ Such mass transfer limitations can be an issue in batch mode conversions, where mostly homogeneous catalytic systems are used.

In contrast to batch mode conversions of CO_2_, in continuous flow chemistry, even higher pressures can be applied under safe conditions. While reactions with normal CO_2_ gas cylinders in batch are typically limited to feed pressures of 5 MPa, continuous conversions can be safely realized with pressures up to 50 MPa. In addition, flow chemistry offers a higher level of automation as well as linear scalability.^51^

Regarding the synthesis of cyclic carbonates, only a few examples of flow conversions are literature-known and summarized in a recent published review.^52^ In this context, our group published in 2018 the synthesis of propylene carbonate under supercritical conditions in continuous flow.^48^

In this paper, we went one step further to a more complex, less reactive, and thus more challenging but also bioderived substrate and presented an optimized long-term conversion of bioderived limonene oxide and limonene dioxide to limonene carbonates in continuous flow. Supercritical carbon dioxide act as a reagent and sole solvent. Easily producible heterogeneous SILP catalysts were applied.

## Results and Discussion

### Selection of Catalysts: Ionic Liquids and SILPs

**Figure 1:**
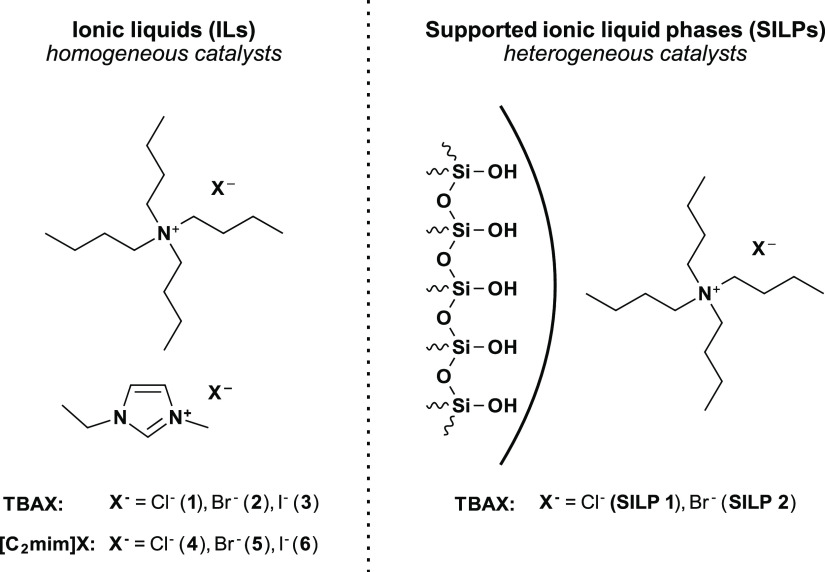
Catalysts: ammonium- and imidazolium-based ionic liquids served as homogeneous catalysts (left), and SILPs, where the ionic liquid was physisorbed on mesoporous silica, were applied as heterogeneous catalysts (right).

So far, Morikawa *et al*.,^30,31^ Mülhaupt *et al.*,^32−34^ and Hintermair *et al.*(35) dealt with the formation of cyclic carbonates starting from limonene oxides using tetrabutylammonium halides as sole catalysts but only under batch conditions. Based on these publications, we chose tetrabutylammonium-based halides (TBAC **1**, TBAB **2**, and TBAI **3**) for catalyst screening in batch mode followed by application in continuous flow. In addition, 1-ethyl-3-methyl imidazolium halides ([C_2_mim]Cl **4**, [C_2_mim]Br **5**, and [C_2_mim]I **6**) were investigated based on our experience in continuous flow conversion of propylene oxide.^48^ An overview of used catalysts is shown in Figure 1.

For the continuous production of limonene carbonates in heterogeneous mode, SILP catalysts (**SILP 1** and **2**, see Figure 1) were prepared according to a general procedure,^48^ where the supporting material and ionic liquid was suspended and dissolved in dichloromethane and shaken for 1 h. After removal of the solvent, a SILP with a thin physisorbed film of ionic liquid on mesoporous silica-60 was obtained. The high surface area of the catalytically active material enables an ideal mass transfer,^50^ which can be an issue in homogeneous catalysis, especially if no solvent is used, which is, on the other side, desirable with regard to sustainable chemistry.

### Batch Conversion of Limonene Oxides: Catalyst Screening and Optimization

**Figure 2:**
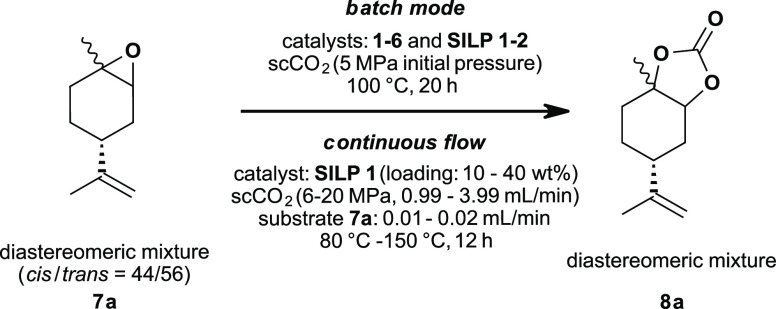
Limonene oxide **7a**: catalyst screening in batch mode followed by the development and optimization of the continuous flow process using heterogeneous SILP catalysts.

We commenced our investigation by screening catalysts and optimizing the synthesis of limonene carbonates **8** in batch mode (Figures 2 and 4).

Initially, we focused on the conversion of limonene oxide **7a** (Figure 2) due to a simple analysis of diastereomeric product mixtures. For the determination of yields, NMR spectroscopy with naphthalene as the internal standard was used.^30^

As shown in Table 1, during the screening of tetrabutylammonium and 1-ethyl-3-methylimidazolium halides **1–6** in batch mode, tetrabutylammonium chloride (TBAC **1**) turned out to be the most selective and highest-yielding catalyst for the conversion of diastereomeric mixture (*cis*/*trans* = 44/56) of limonene oxide **7a** to the corresponding limonene carbonate **8a** (entry 1). Furthermore, no byproducts were formed, as proven by NMR and GC/MS measurements.

**Table 1:** Limonene Oxide **7a**: Results of Catalyst Screening in Batch Mode

		conversion of isomer **7a** (NMR) [%]^a^			yield of **8a** (NMR) [%]^a^
entry	catalyst	*cis*	*trans*	sum	sum
1	TBAC **1**	43 (60^b^)	94 (100^b^)	72 (83^b^)	68 (57^c^, 50^d^)
2	TBAB **2**	47	76	63	56 (32^d^)
3	TBAI **3**	25	35	31	12 (7^d^)
4	[C_2_mim]Cl **4**	17	19	18	2
5	[C_2_mim]Br **5**	6	17	13	6
6	[C_2_mim]I **6**	11	11	11	0
7^e^	TBAC **1** + silica gel 60	48	84	68	46
8^e^	TBAB **2** + silica gel 60	71	71	71	29
9^e^	TBAI **3** + silica gel 60	69	70	70	29
10^e^	[C_2_mim]Cl **4** + silica gel 60	12	22	17	7
11^e^	[C_2_mim]Br **5** + silica gel 60	37	30	33	11
12^e^	[C_2_mim]I **6 +** silica gel 60	75	49	61	10
13	**SILP 1** (20 wt % TBAC **1**)	44	78	63	62
14	**SILP 2** (20 wt % TBAB **2**)	50	63	57	33

a Conditions: 5 MPa CO_2_ (gaseous, initial pressure), 5 mmol limonene oxide **7a** (*cis/trans* = 43/57), 10 mol % catalyst **1–6**, 13 mg of naphthalene (internal standard), 100 °C, 20 h. Further information about the calculations of NMR yields are summarized in the Supplementary Information (ESI Figure S12 and Formulas S1–S6).

b Conversions after 70 h.

c Isolated yield after column chromatography.

d Published values from ref (30) (conditions according to table note a except CO_2_ pressure [a lower CO_2_ pressure of 3 MPa and dry ice were used]).

e Conditions according to table note a, 10 mol % catalyst **1–6** (20 wt %), and silica gel 60 (80 wt %).

As already shown by kinetic studies in the literature,^53^ an increase in pressure of CO_2_ from 3 to 5 MPa led to higher yields up to 24% as in the case of TBAB **2** (entry 2). Nevertheless, compared to TBAC **1** (entry 1), TBAB **2** (entry 2) showed a slight decrease in yield and selectivity. The order of reactivity of Cl^–^ > Br^–^ > I^–^ is in accordance to the expected nucleophilicity of halides in polar aprotic reaction environments (entries 1–3). The imidazolium-based ionic liquids **4–6** were found to be catalytically less or even inactive (entries 4–6). This is in contrast to our previous studies, where imidazolium-based catalysts where identified as more suitable.^48^ Regarding steric effects, the sterically more demanding *cis* isomer of limonene oxide **7a** showed in all cases a lower conversion than the *trans* isomer. After 20 h, TBAC **1** (entry 1) resulted in a conversion of 94% of the *trans* isomer and 43% of the *cis* isomer (in total 72%) and an overall yield of carbonate **8a** of 68%. Purification via column chromatography resulted in 57% isolated yield. Furthermore, after 70 h at 100 °C, the *cis* isomer showed 60% conversion, whereas the *trans* isomer indicated full conversion.

For the screening of the catalysts in the presence of silica without immobilization (entries 7–12), TBAC **1** (entry 7) and TBAB **2** (entry 8) showed a lower yield and lower selectivity. For TBAI **3** (entry 9) and the imidazolium-based catalysts **4–6** (entries 10–12), the yields increased slightly; nevertheless, the selectivities remained in the lower range.

The catalyst screening of supported ionic liquid phases (**SILP 1** and **SILP 2**) of ammonium-based ionic liquids **1**–**2** physisorbed on silica resulted in the same order regarding the catalytic activity than in homogeneous mode (entries 13 and 14). **SILP 1** (entry 13) with immobilized TBAC **1** gave again the highest yield and selective conversion to the desired carbonate **8a**.

Recycling studies of **SILP 1** (see ESI Table S3) revealed that the yield decreased from 62 to 50% after the first recycling step and leveled at 25% after the fourth cycle. **SILP 2** was recyclable for three times (see ESI Table S3) without a significant change in yield from 31 to 29%; after the fourth cycle, the yield slowly decreased from 29 to 23%. Nevertheless, the yield as well as selectivity (entries 13 and 14) was generally lower compared to **SILP 1**. For this reason, **SILP 1** was used for further studies.

As a general side reaction in the presence of silica, the ring opening of the epoxide^54^ to limonene diol, catalyzed by the acidic hydroxy groups of silica and residual water in the silica, has to be considered. The influence of water was proven by the addition of 10 wt % of water (entry S2) to the reaction mixture where 20% of limonene diol was formed according to GC/MS. In batch mode using **SILP 1** (entry 13) as the catalyst, 5% of limonene diol was formed according to GC/MS. However, the diol was no longer formed in continuous flow, which can be explained by a shorter interaction of the substrate and supported catalyst in continuous flow than in batch mode.

Further studies on the optimization of the SILP system (see ESI Table S2) revealed that the free hydroxy groups of silica had a beneficial effect on the reaction. Upon comparing the yields of non-calcined with calcined silica, a 17% lower yield in the case of calcined silica was obtained (entries S11 and S12). This synergistic effect of surface hydroxy groups of supporting materials and ionic liquids in connection with the synthesis of cyclic carbonates is also described in the literature.^55−57^

Furthermore, a decrease in catalyst loading (entries S7–S9) from 20 to 15 or 10 wt % resulted in a drop of yield, and an increase in catalyst loading to 40 wt % (entry S10) gave only a minor increase in yield from 62 to 68%, which can be explained by a fully covered surface and less hydroxy groups that exhibit the mentioned synergistic effect.^55,56^

**Figure 3:**
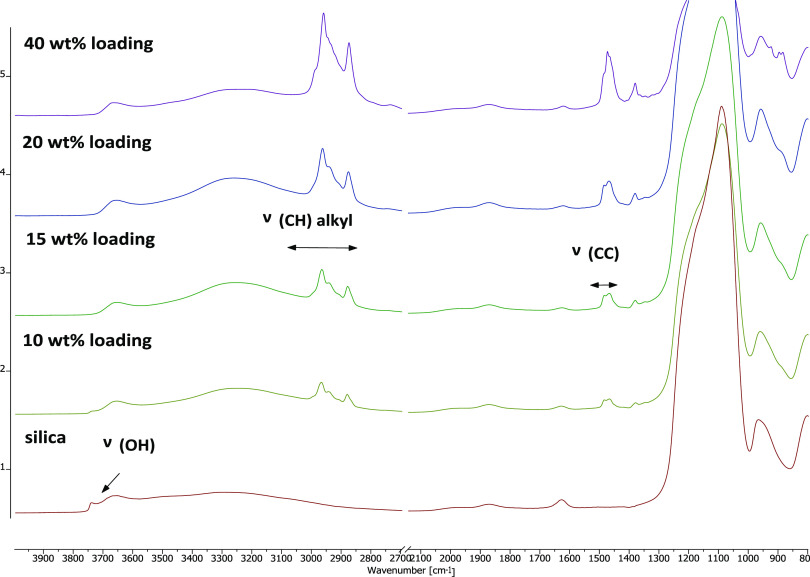
Different catalyst loadings of **SILP 1**: a decrease in surface hydroxy groups (3750 cm^–1^) while increasing the loadings was observed.

The decrease in hydroxy groups on the surface of the material while increasing the catalyst loading from 10 to 40 wt % was also shown by DRIFT spectroscopy, where the band at 3750 cm^–1^ corresponds to the surface hydroxy groups. Apart from that, bands at around 2900 and 1550 cm^–1^ represent the CH and CC vibrations of TBAC **1** (Figure 3), respectively.

The catalyst loadings were also confirmed by TGA measurements (see ESI Figures S5 and S7), where the mass loss of the SILP materials during heating up from 25 to 450 °C with a rate of 5 °C/min was detected. The initial mass loss at around 100 °C was caused by adsorbed water in the SILP material.

Finally, 20 wt % catalyst loading as ideal conditions was chosen for further studies in continuous flow.

**Figure 4:**
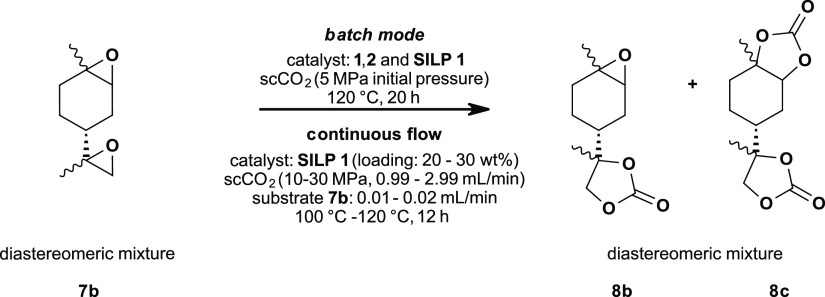
Limonene dioxide **7b**: catalyst screening in batch mode followed by the development and optimization of the continuous flow process using heterogeneous SILP catalysts.

Based on our results of limonene oxide **7a** and the work of Mülhaupt *et al*.^33^ we further expanded our research towards the batch conversion of limonene dioxide **7b** in homogeneous and heterogeneous mode with ammonium based ionic liquids as catalysts (Figure 4). For this reason, we selected TBAC **1**, TBAB **2**, and **SILP 1** as catalysts as they showed the highest activity in the case of limonene oxide **7a** (Table 1, entries 1, 2, and 13).

Yields were determined via GC using octane as the internal standard since NMR analysis was not suitable due to the formation of four diastereomers of epoxycarbonate **8b** and two diastereomers of biscarbonate **8c** leading to overlapping signals.

In the case of all tested catalysts TBAC **1**, TBAB **2**, and **SILP 1** (Table 2), limonene dioxide **7b** was fully converted to carbonate **8b** or **8c**. TBAC **1** showed again the best performance regarding yield and selectivity (entry 15).

**Table 2:** Limonene Dioxide **7b**: Results of Catalyst Screening in Batch Mode

		yield (GC) [%]^a^		
entry	catalyst	**8b**	**8c**	sum (**8b** + **8c**)
15	TBAC **1**	30 (26^b^)	69 (66^b^)	99 (92^b^)
16	TBAB **2**	44	35	79
17	**SILP 1** (20 wt % TBAC **1**)	36	40	76

a Conditions: 5 MPa CO_2_ (gaseous, initial pressure), 1.07 mmol limonene dioxide **7b**, 10 mol % catalysts **1** and **2**, 120 °C, 20 h. Further information regarding the determination of GC yields is shown in the Supplementary Information (ESI Figures S17 and S18).

b Isolated yield after column chromatography.

Using TBAC **1** (entry 15) as a homogeneous catalyst, a total yield of 99% was obtained after 20 h at 120 °C; hence, 30% of epoxycarbonate **8b** and 69% of biscarbonate **8c** were formed. Additionally, full conversion to biscarbonate **8c** was observed after 67 h at 120 °C.

As a side reaction in heterogeneous catalysis with **SILP 1** (entry 17), the formation of limonene diol via acid ring opening^54^ of up to 13% was observed (verified via GC/MS). Nevertheless, the diol was not formed in continuous flow experiments for reasons already discussed for limonene oxide **7a**.

With this SILP system and determination of yields via GC in hand, several continuous flow experiments were conducted in the following.

### Continuous Flow Conversion of Bioderived Limonene Oxides to Various Limonene Carbonates

#### General Setup for Continuous Flow Reactions

**Figure 5:**
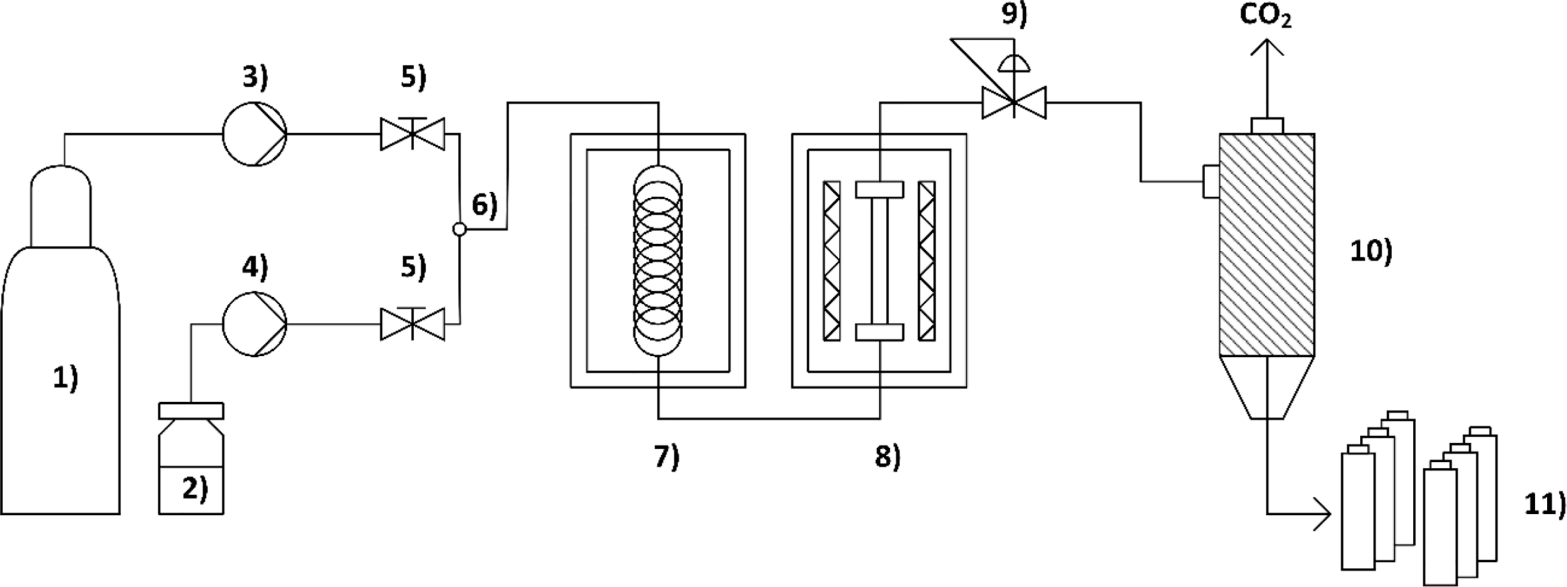
Schematic representation of the scCO_2_ flow device: (1) liquid CO_2_ supply, (2) substrate supply, (3) CO_2_ pump, (4) substrate pump, (5) hand operated valve, (6) T-piece, (7) oven with preheating coil (up to 80 °C), (8) oven with catalyst cartridge (up to 150 °C), (9) back-pressure regulator, (10) gas–liquid separator, and (11) product collector.

All continuous flow reactions were performed with the following reaction setup shown in Figure 5. A CO_2_ cylinder with an ascending pipe served as the gas supply, CO_2_ was pumped through the system with an HPLC pump. A glass vial, filled with the corresponding limonene oxide **7a** or **7b**, served as the substrate supply and was pumped through the system with an HPLC pump. Experiments with flow rates down to 0.01 mL/min were performed since lower flow rates are not recommended for reasons of accuracy of the used HPLC pumps.

As already discussed for the batch mode, no co-solvent was used, which is advantageous with regard to sustainability and leaching of the catalyst, especially in long-term experiments. CO_2_ and the substrate were mixed before entering a thermostated unit where the catalyst cartridge filled with SILP materials was located. In the case of temperatures higher than 80 °C, the substrate/scCO_2_ mixture was preheated in a coil to 80 °C before entering a second heating unit where the catalyst cartridge could be heated up to 150 °C. Catalyst cartridges of two different lengths were used (150 and 250 mm) during optimization, resulting in different catalyst input (1.34 and 2.22 g of SILP material). In order to perform reactions at different pressures, a back-pressure regulator was involved in the system. After passing the gas–liquid separator, the product was collected as a mixture of carbonates **8** and unreacted starting material **7** excluding any byproduct, as verified by NMR and GC/MS measurements. Further technical details of the setup are provided in the Materials and Methods section.

Conversion and yields of the sampled product **8** were determined in the case of limonene oxide **7a** via NMR analysis^30^ (internal standard: naphthalene) and in the case of limonene dioxide **7b** via GC (internal standard: octane) as described above. Further information on the determination of yields are given in the Supplementary Information (ESI sections 4.1 and 5.1). Leaching of ionic liquid from the supporting material was quantified via ^1^H-NMR spectroscopy using naphthalene as the internal standard.

#### Continuous Flow Conversion of Limonene Oxide 7a

As shown in Figure 2, the optimization of the continuous flow conversion of limonene oxide **7a** to limonene carbonate **8a** included variation of flow rates for the substrate and CO_2_, catalyst loading on the SILP material, pressure, and temperature.

**Figure 6:**
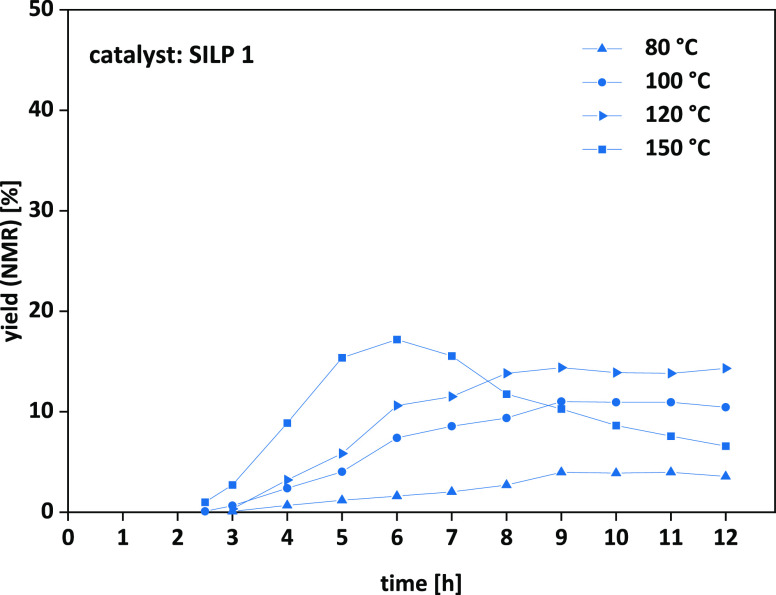
Limonene oxide **7a**: temperature screening in continuous flow. Detailed conditions are given in Table 3.

Following the results from the batch mode experiments and our experience from a previous project with propylene oxide,^48^ a flow rate of 1.99 mL/min for CO_2_ and 0.01 mL/min for limonene oxide **7a**, 1.34 g of **SILP 1** (150 mm length of catalyst cartridge, residence time: 75 s) with a catalyst loading of 20 wt % TBAC **1**, and a pressure of 10 MPa were chosen as the starting point for a temperature screening (Figure 6 and Table 3, entries 18−21).

**Table 3:** Limonene Oxide **7a**: Influence of Temperature, Pressure Catalyst Input, and Catalyst Loading in Continuous Flow Using **SILP 1** as a Heterogeneous Catalyst^a^

					yield (NMR)^b^ [%]		
entry	temperature [°C]	pressure [MPa]	input **SILP 1** [g]	catalyst [wt %]	maximum	overall (12 h)	leaching^c^
18	80	10	1.34	20	4	2	n.o.
19	100				11	6	<1%
20	120				14	8	<1%
21	150				17	9	degradation^d^
							
22	120	6	1.34	20	12	6	12%
23		10			14	8	<1%
24		15			12	8	n.o.
25		20			11	7	n.o.
							
26	120	15	2.22	20	19	12	n.o.
27		15		30	22	15	n.o.
28		15		40^e^	26	16	<1%

a Reactions were carried out with **SILP 1** using catalyst cartridges of 150 mm (1.34 g of **SILP 1**, residence time: 75 s) or 250 mm length (2.22 g of **SILP 1**, residence time: 125 s) under the following conditions: flow rate of limonene oxide **7a** (*cis/trans* = 43/57): 0.01 mL/min, flow rate CO_2_: 1.99 mL/min, 12 h.

b Yields are given as sum of the *cis* and *trans* isomer. Internal standard: naphthalene. Further information about the calculations of NMR yields are summarized in the Supplementary Information (ESI Figure S12 and Formulas S1–S6).

c For determination of leaching, the integral of the signal at δ = 3.35 ppm of TBAC **1** was used (limit of detection: 0.5–1 mg; ≤0.2%).

d The Hoffmann elimination product was obtained.

e Inconstant product output due to overpressure during the reaction.

As shown in Figure 6, the reaction started after 2–3 h preliminary lead time, which is in accordance to low flow rates of limonene oxide **7a** of 0.01 mL/min. Increasing the temperature from 80 °C up to 120 °C (entries 18–20) resulted in increasing and constant outputs (maximum yield: 14%) of limonene carbonate **8a**. In contrast, at 150 °C (entry 21), Hoffmann elimination of TBAC **1** to tributylamine became an issue, resulting in a decrease in yield over time. The formation of the elimination product was confirmed via ^1^H-NMR analysis. Additionally, the observed thermal stability is in accordance with thermogravimetric analysis data, where degradation also started at around 150 °C (see ESI Figure S6).

Besides thermal stability, preventing leaching of the catalyst from the supporting material is of high importance especially with regard to industrial applications and long-term use of the catalytic system. In this context, during the temperature screening (entries 18–21) performed at 10 MPa, either no leaching or values below 1% of leached TBAC **1** were detected via NMR spectroscopy (limit of detection: 0.5–1 mg; ≤0.2%).

While screening pressures from 6 to 20 MPa (entries 22–25), it turned out that a pressure of 6 MPa (entry 22) led to leaching of 12%. In contrast, at operating pressures of 15 and 20 MPa (entries 24 and 25), no leached catalyst was detected via NMR spectroscopy. Additionally, 15 MPa (entry 24) was found to be the optimum pressure because at higher pressures, a trend to decreased yields was observed (entry 25).

In order to increase the yields, a catalyst cartridge of 250 mm (residence time: 125 s) instead of 150 mm length (residence time: 75 s) was used, resulting in a 65% higher input of SILP and equally longer residence time. With the increase in the input of SILP to 2.22 g, an increase of 60% in maximum yield from 12 to 19% was observed (entries 24 and 26). Additionally, a constant output over 12 h was achieved.

An increased catalyst loading from 20 to 30 wt % (entries 26 and 27) resulted in a slight increase in yield to 22% maximum yield and 15% overall yield paired with completely suppressed leaching of the catalyst. However, a further increase to 40 wt % catalyst loading (entry 28) ended up in an overpressure in the system during the reaction due to a visible agglomeration and loss in the free-flowing property of the SILP material.

As a last step in optimization, the impact of different flow rates of CO_2_ and substrate on the so far optimized system (entry 27; 2.22 g of **SILP 1**, 30 wt % loading, 15 MPa, 120 °C) was studied (see ESI Figure S13 and Table S4).

Flow rates of CO_2_ between 1.99 mL/min (residence time: 125 s, entry S15) and 2.49 mL/min (residence time: 100 s, entry S16) resulted in overall yields of 15–16% as well as no leaching of the catalyst and thus turned out to be the optimum. With a higher flow rate of 3.99 mL/min (residence time: 62 s, entry S17), lower overall yields of 12% were achieved due to a shorter residence time. In contrast, a lower flow rate of 0.99 mL/min (residence time: 250 s, entry S13) led to a blockage of the flow device and therefore a non-constant product output. This also confirmed the necessity of the solvent environment provided by scCO_2_ as the sole solvent.

In order to further increase productivity of the process, the double flow rate of limonene oxide **7a** (0.02 mL/min, entry S18) was applied to the system. However, a higher flow rate led to leaching of the catalyst and therefore to a decrease in yield over time.

**Figure 7:**
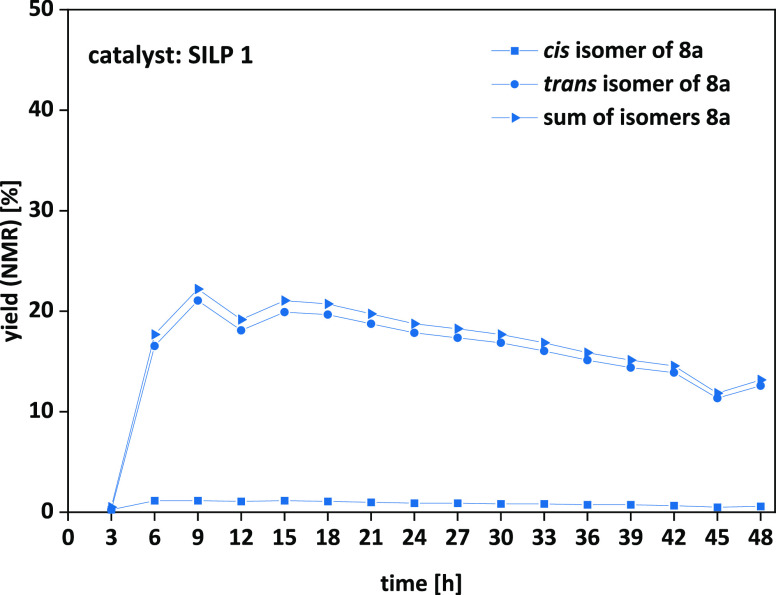
Limonene oxide **7a**: long-term stability of **SILP 1** over 48 h. Final optimized conditions: **SILP 1** (2.22 g, 30 wt % loading), 1.99 mL/min CO_2_, 0.01 mL/min limonene oxide **7a**, 15 MPa, 120 °C, 48 h, 250 mm catalyst cartridge.

With the optimized conditions in hand (entry 27; **SILP 1** (2.22 g, 30 wt % of TBAC **1**), 1.99 mL/min CO_2_, 0.01 mL/min **7a**, 15 MPa, 120 °C, 250 mm catalyst cartridge), the long-term stability of our catalytic system over 48 h was further investigated (Figure 7).

The long-term stability studies of the *cis*/*trans* mixture of limonene oxide **7a** with **SILP 1** as the catalyst over 48 h resulted in a maximum yield of 22% and an overall yield of 16%, respectively, with a production rate of 0.12 g/h of pure limonene carbonate **8a** dissolved in starting material **7a**. However, taking the ratio of the *cis* and *trans* isomer of 43/57 as well as the low reactivity of the *cis* isomer into account, the yield can be further increased by performing continuous flow reactions exclusively with the more reactive *trans* isomer as already investigated in batch mode.^31^ Nevertheless, in order to cover the reactivity of both isomers, the commercially available *cis*/*trans* mixture of limonene oxide **7a** was used for these purposes.

Additionally, no leaching over 48 h (Figure 7) as well as over 96 h (ESI Figure S14) was observed according to NMR analysis of the product fractions (limit of detection: 0.5–1 mg; ≤0.2%). For further studies on the catalyst stability, the recovered SILP was dried under vacuum and leached with methanol followed by NMR analysis showing no degradation of TBAC **1**.

The slight decrease in yield over time can be explained by agglomeration of the starting material, product, and intermediate on the catalytically active surface as shown by N_2_ physisorption measurements (*vide infra*).

#### Continuous Flow Conversion of Limonene Dioxide **7b**

Based on the results of the optimization of limonene oxide **7a**, we ultimately addressed the conversion of limonene dioxide **7b**, aiming for selective formation of diastereomeric mixtures of epoxycarbonate **8b** and biscarbonate **8c**. The temperature, pressure, catalyst loading, and the flow rates of CO_2_ and limonene dioxide **7b** were varied (Figure 4); results of the optimization are shown in Table 4.

**Table 4:** Limonene Dioxide **7b**: Influence of Temperature, Pressure and, Catalyst Loading in Continuous Flow Using **SILP 1** as the Heterogeneous Catalyst^a^

				yield (GC)^b^ [%]				
				maximum		overall (12 h)		
entry	temperature [°C]	pressure [MPa]	catalyst loading [wt %]	**8b**	**8c**	**8b**	**8c**	sum
29	100	15	30	23	10	14	5	19
30	120			30	18	18	8	26
31	120	10	30	52	22	21	6	27
32		15		30	18	18	8	26
33		20		27	14	17	8	25
34		30		22	10	15	6	22
35	120	20	20	26	7	16	4	20
36			30	27	14	17	8	25

a Reactions were carried out with **SILP 1** using a catalyst cartridge of 250 mm length (2.22 g of **SILP 1**, residence time: 125 s) under the following conditions: flow rate of limonene dioxide **7b**: 0.01 mL/min, flow rate of CO_2_: 1.99 mL/min (2× 0.995 mL/min), 12 h.

b Internal standard: octane. Further information regarding the determination of GC yields is shown in the Supplementary Information (ESI Figures S17 and S18).

Independent from the used parameters (Table 4, entries 29–36), the yield of epoxycarbonate **8b** was higher than biscarbonate **8c**, which can be explained by differing steric hindrance and therefore reactivity of the epoxide groups of limonene dioxide **7b**.

During the screening, a temperature of 120 °C was found to be the optimum temperature (entry 30). Maximum yields of 30% for epoxy carbonate **8b**, 18% for biscarbonate **8c**, and an overall yield of 26% were achieved in a constant output of product over 12 h. A lower temperature of 100 °C (entry 29) resulted in a lower overall yield of 19%; higher temperatures were not suitable according to the thermal stability of **SILP 1** as already shown for limonene oxide **7a** (Figure 6).

**Figure 8:**
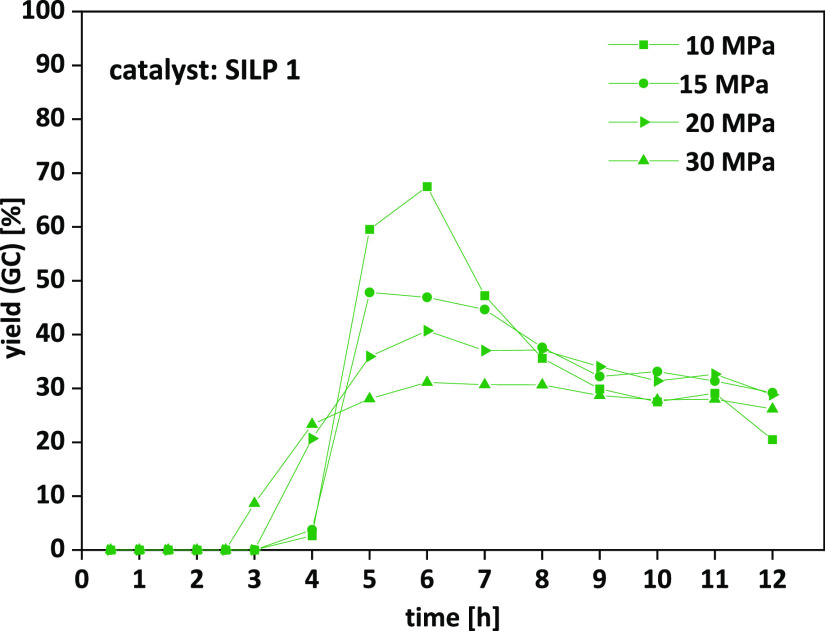
Limonene dioxide **7b**: pressure screening in continuous flow. Detailed conditions are given in Table 4.

During screening of different pressures (entries 31–34), the highest overall yield of 27% was achieved at 10 MPa (entry 31); however, pressures of 15–30 MPa (entries 32–34) resulted in a higher constancy of product output over time (Figure 8), which is of higher interest in continuous flow chemistry than having high yields for a short period of time. In terms of overall yield, 15 MPa (entry 32) and 20 MPa (entry 33) turned out to be the best conditions (Table 4).

Increasing the catalyst loading from 20 to 30 wt % (entries 35 and 36), an increase in overall yield from 20 to 25% was achieved. The increase in the maximum yield of biscarbonate **8c** from 7 to 14% has also to be mentioned at this point. A higher catalyst loading of 40 wt % was not suitable according to inconstant product outputs in the case of limonene oxide **7a** (Table 3, entry 28) being caused by visible agglomeration and loss of free-flowing property of the SILP material.

Flow rates of CO_2_ (Table 5) in a range of 0.99–3.99 mL/min (entry 37–39) resulted in constant outputs of carbonates **8b** and **8c**. However, with a flow rate of 1.99 mL/min CO_2_ (entry 38), the best overall yield of 25% and maximum yields of 27% for epoxycarbonate **8b** and 14% for biscarbonate **8c** were obtained. The trends to lower yields when higher flow rates of CO_2_ are applied are in accordance with a decrease in the residence time of the substrate on the catalyst.

**Table 5:** Limonene Dioxide **7b**: Influence of Flow Rates of CO_2_ and Substrate in Continuous Flow Using **SILP 1** as the Heterogeneous Catalyst^a^

				yield (GC)^b^ [%]				
	flow rates [mL/min]							
entry	CO_2_	substrate **7b**		maximum		overall (12 h)		
			residence time [s]	**8b**	**8c**	**8b**	**8c**	sum
37	0.99	0.01	250	31	18	17	7	24
38	1.99		125	27	14	17	8	25
39	3.99		62	21	8	13	5	17
40	1.98	0.02	125	21	7	13	4	17

a Reactions were carried out with **SILP 1** using a catalyst cartridge of 250 mm length (2.22 g of **SILP 1**) under the following conditions: 20 MPa, 120 °C, 12 h.

b Internal standard: octane. Further information regarding the determination of GC yields is shown in the Supplementary Information (ESI Figures S17 and S18).

A higher flow rate of limonene dioxide **7b** (Table 5) of 0.02 mL/min (entry 40) resulted in a drop of overall yield from 25 to 17% caused by leaching of the immobilized catalyst over time.

**Figure 9:**
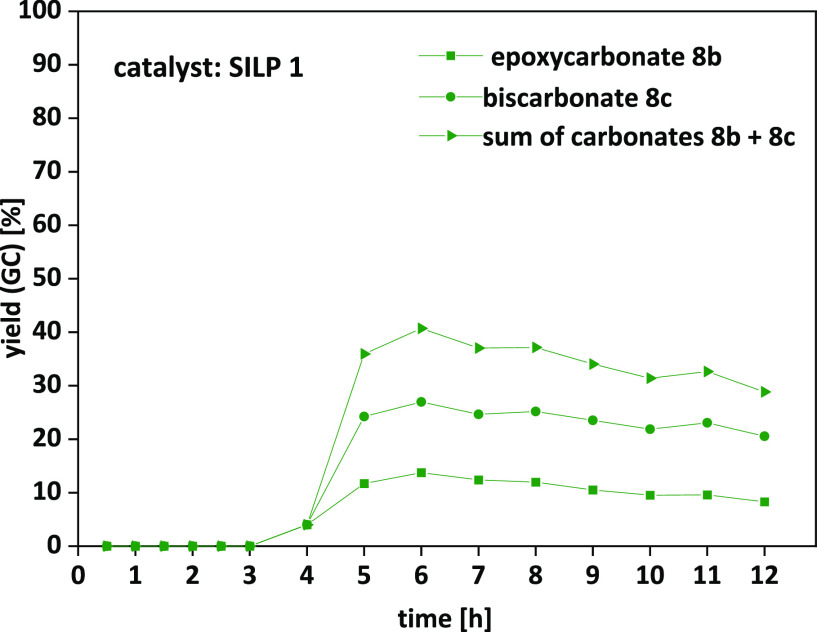
Limonene dioxide **7b**: optimized conditions resulted in an output of carbonates **8b** and **8c** of overall 25%. Final optimized conditions: **SILP 1** (2.22 g, 30 wt % of TBAC **1**), 1.99 mL/min CO_2_, 0.01 mL/min limonene dioxide **7b**, 20 MPa, 120 °C, 12 h, 250 mm catalyst cartridge.

Hence, with the optimized conditions (Figure 9), maximum yields of 27% for epoxycarbonate **8b** and 14% for biscarbonate **8c** and an overall yield of 25% were obtained. The slight decrease in yield over time was caused by leaching of catalyst from the supporting material, which is also visible in long-term stability experiments (see ESI Figure S19).

The long-term experiment over 48 h of **SILP 1** with a catalyst loading of 30 wt % resulted in an overall yield of 16% (11% of **8b** and 5% of **8c**). However, leaching of 50% of immobilized TBAC **1**, most dominantly in the first 9 h, was observed, resulting in a decrease in yield over time.

For this reason, the catalyst loading was reduced to 15 wt %, whereas the overall yield of 17% remains unchanged. Furthermore, only traces of leached catalyst were detected via ^1^H-NMR spectroscopy (limit of detection: 0.5–1 mg; ≤0.2%) in the fractions of the first 27 h. After 27 h, no leaching and a constant output of carbonates **8b** and **8c** were observed, resulting in an overall yield of 17% over 48 h and a production rate of 0.13 g/h.

Overall, by reducing the catalyst loading from 30 to 15 wt %, leaching was suppressed almost completely, reflecting in a product output of 17% overall yield.

Measurements of the surface area and porosity via N_2_ physisorption confirmed that, apart from leaching, the loss in yield was caused by the proceeding agglomeration of the starting material, product, or intermediate on the catalytically active surface over time. The characterization of the SILP catalysts via N_2_ physisorption using the Brunauer–Emmett–Teller (BET) as well as the Barrett–Joyner–Halenda (BJH) method revealed that the surface area of the SILP catalyst dropped significantly from 451 to 231 m^2^/g (reference material silica gel 60: 634 m^2^/g) after 48 h of reaction time compared to the freshly prepared SILP catalyst. In addition, the decrease in pore volume from 0.57 to 0.34 cm^3^/g (reference material silica gel 60: 0.91 cm^3^/g) and the average pore diameter from 49.07 to 45.40 Å clearly reflected this trend (see ESI Table S1 and Figure S11).

## Conclusions

We developed a continuous flow method for the selective synthesis of three different bioderived carbonates **8a–c** starting from limonene oxide **7a** and limonene dioxide **7b**. Thereby, supercritical carbon dioxide (scCO_2_) served as the reactant and sole solvent. Ammonium- and imidazolium-based halides as ionic liquid catalysts were screened in batch mode, tetrabutylammonium chloride TBAC **1** turned out to be a high-yielding and a selective catalyst. The SILP concept (supported ionic liquid phase) was used for immobilization of ionic liquid **1** on silica followed by applying the SILP catalyst **SILP 1** in heterogeneous continuous flow mode. After optimizing the continuous flow parameters (temperature, pressure, flow rates, and catalyst loading) for both limonene oxides **7** in 12 h experiments, the catalytic system was successfully studied in long-term experiments over 48 h, eventually providing a constant product output with 16–17% yield.

Ultimately, SILPs in combination with scCO_2_ were confirmed as an easily obtained and highly suitable combination for continuous flow chemistry, although yields in this particular example remained in the lower range. Our future studies will address the development of more reactive catalysts, focusing in particular on different cationic cores. In this regard, work is currently ongoing in our group.

As an outlook, a scaled flow process for limonene carbonates as potential bioderived bulk chemicals with production rates in the range of kilograms per hour is of particular interest. In this regard, a setup suitable for higher flow rates of carbon dioxide and limonene oxides as well as for bigger catalyst cartridges for SILP catalysts is expected to be crucial.

## Experimental Part

### Materials and Methods

More information on used materials and methods are summarized in the Supplementary Information (ESI section 1).

Continuous flow experiments were performed with a scCO_2_ continuous flow device from Jasco (Jasco Corporation, Tokyo, Japan). CO_2_, provided by Messer Austria GmbH (>99.995% purity; with ascension pipe), was cooled to −7 °C by a recirculating cooler (CF 40, JULABO GmbH) and was introduced by two CO_2_ pumps (PU-2086Plus) with cooled heads. An HPLC pump (PU-2089Plus) delivered substrates. Catalyst cartridges (empty 316 stainless-steel HPLC columns from Restek; 150 mm × 4.6 mm × 1/4″ OD, 2 μm frits, 2.49 mL volume, and 1.34 g of SILP catalyst and 250 mm × 4.6 mm × 1/4″ OD, 2 μm frits, 4.15 mL volume, and 2.22 g of SILP catalyst) filled with SILP catalysts (the maximum weight of packing is dependent on catalyst loading; silica gel 60 served as the reference material for the determination of weight of packing) were heated up in an HPLC column oven (CO-2060Plus, up to 80 °C; Brinkmann CH-500 HPLC column heater system, up to 150 °C). A back-pressure regulator (BP-2080Plus, temperature set to 60 °C), UV detector (UV-2075Plus), and a product collector (SCF-Vch-Bp) were also included and were all connected with 1/16″ stainless-steel tubings.

### Preparation of Supported Ionic Liquid Phases on the Example of **SILP 1** (30 wt % of TBAC **1**)

The syntheses and analytical data of the ionic liquids are summarized in the Supplementary Information (ESI section 2).

SILPs were prepared according to a modified literature procedure.^48^ Tetrabutylammonium chloride **1** (7.000 g, 30 wt %), dried under high vacuum overnight, was dissolved in 100 mL of dry dichloromethane. Silica gel 60 (21.000 g, 70 wt %), dried in a vacuum oven (50 °C, 50 mbar) for 3 days, was added to the solution. The suspension was shaken for 60 min at 480 rpm. The solvent was removed *in vacuo* followed by further drying under high vacuum. TGA analysis and DRIFT spectra are given in the Supplementary Information (ESI section 3).

### Conversion of Limonene Oxide **7a** under Batch Conditions on the Example of TBAC **1** and **SILP 1** as Catalysts

The formation of limonene carbonate **8a** under batch conditions was performed according to a modified literature procedure.^30^ Limonene oxide **7a** (*cis*/*trans* = 43/57, 761 mg, 5.00 mmol, 1.00 equiv) and naphthalene as the internal standard (13 mg, 0.10 mmol) were mixed together. A ^1^H-NMR spectrum (*t* = 0) was measured as the reference (see also Figure S12).

A 40 cm^3^ stainless-steel autoclave was charged either with TBAC **1** (139 mg, 0.50 mmol, 10 mol % with respect to the epoxide) or with **SILP 1** (695 mg, 20 wt % of TBAC **1**, 0.50 mmol TBAC **1**, respectively, 10 mol % TBAC **1**), the previously prepared mixture, and CO_2_ (5 MPa). The reaction mixture was stirred at 100 °C for 20 h. After 20 h, the autoclave was cooled to room temperature and CO_2_ was released. In the case of heterogeneous catalysis, the crude mixture was diluted with 5 mL of deuterated chloroform and homogenized. For the determination of the yield, a ^1^H-NMR spectrum (*t* = 20) of the crude mixture was recorded (see Figure S11). For verifying the NMR yield, the isolation was performed once via column chromatography (LP:EA = 10/1–1/1, 50 g of silica). catalyst TBAC **1**: NMR yield: 68% (isolated: 57%, colorless oil); catalyst **SILP 1**: NMR yield: 62%, FTIR (ATR, neat): 2942 (alkyl), 1790 (C=O) cm^–1^; ^1^H-NMR (600 MHz, CDCl_3_ CH_4_Si): δ 4.74 (t, J = 1.6 Hz, *cis* 1H), 4.72 (t, J = 1.5 Hz, *trans* 1H), 4.70–4.68 (m, *cis* 1H + *trans* 1H), 4.43–4.40 (m, *cis* 1H), 4.35 (dd, J = 9.5, 7.0 Hz, *trans* 1H), 2.30–2.18 (m, *cis* 2H + *trans* 2H), 2.02–1.94 (m, *cis* 1H), 1.94–1.85 (m, *trans* 1H), 1.84–1.74 (m, *cis* 2H), 1.70 (s, *cis* 3H), 1.68 (s, *trans* 3H), 1.67–1.55 (m, *cis* 1H + *trans* 2H), 1.49–1.47 (m, *cis* 3H), 1.45–1.34 (m, *trans* 5H), 1.24–1.08 (m, *cis* 1H). ^13^C NMR (101 MHz, CDCl_3_, CH_4_Si): δ 154.87 (*trans*), 154.61 (*cis*), 147.53 (*cis*), 147.42 (*trans*), 110.27 (*trans*), 110.05 (*cis*), 82.78 (*cis*), 82.24 (*trans*), 81.93 (*cis*), 80.66 (*trans*), 40.01 (*trans*), 37.42 (*cis*), 34.26 (*cis*), 34.07 (*trans*), 33.14 (*trans*), 30.66 (*cis*), 26.36 (*cis*), 26.27 (*trans*), 25.77 (*trans*), 22.35 (*cis*), 20.99 (*cis*), 20.66 (*trans*) ppm.

### Conversion of Limonene Dioxide **7b** under Batch Conditions on the Example of TBAC **1** and **SILP 1**

The batch reactions were performed according to a modified literature procedure.^33^ An 8 mL glass vial, charged either with TBAC **1** (31 mg, 0.11 mmol, 10 mol %) or with **SILP 1** (83 mg, 30 wt % TBAC **1** loading, 0.11 mmol TBAC **1**, 10 mol % TBAC **1**), limonene dioxide **7b** (180 mg, 1.07 mmol, 1.00 equiv), and CO_2_ (5 MPa), was placed in a 40 cm^3^ stainless-steel autoclave. The reaction mixture was stirred at 120 °C for 20 h.

After 20 h, the autoclave was cooled to room temperature and CO_2_ was released. For verifying the GC yield, the isolation of products was performed once via column chromatography (LP:EA = 6:4, 15 g of silica).

For the determination of the GC yield, the crude mixture was homogenized with 5 mL of ethyl acetate (36 mg limonene dioxide /mL). An aliquot of 42 μL of crude solution, 30 μL of internal standard (20 mg octane/mL ethyl acetate), and 1428 μL of ethyl acetate resulted in a 1.5 mL GC sample. The identity of the peaks was verified via GC/MS.

Catalyst TBAC **1**: GC yield: 99% of carbonates **8b** and **8c** (**8b**: 30%, isolated: 26%; **8c**: 69%, isolated: 66%; colorless oils), **SILP 1** (30 wt % TBAC **1**): GC yield: 76% (**8b**: 36%, **8c**: 40%); epoxycarbonate **8b** (diastereomeric mixture, 94:3:2:1), FTIR (ATR, neat)**:** 2932 (alkyl), 1778 (C=O) cm^–1^; ^1^H-NMR (400 MHz, CDCl_3_): δ 4.23 (dd, J = 8.5, 2.7 Hz, 1H), 4.13–3.96 (m, 1H), 3.31–3.01 (m, 1H), 2.33–2.07 (m, 1H), 2.06–1.73 (m, 3H), 1.71–1.53 (m, 2H), 1.43 (s, 3H), 1.32 (s, 3H), 1.21–1.01 (m, 1H). ^13^C NMR (101 MHz, CDCl_3_): δ 154.62, 154.60, 85.34, 85.37, 73.53, 73.25, 60.06, 60.04, 57.56, 57.35, 37.73, 37.53, 28.71, 28.62, 26.53, 26.31, 24.32, 24.27, 22.29, 22.12, 22.11, 21.99 ppm. Biscarbonate **8c** (diastereomeric mixture, 34:66), FTIR (ATR, neat): 2983 (alkyl), 1775 (C=O) cm^–1^; ^1^H-NMR (400 MHz, CDCl_3_, CH_4_Si): δ 4.55–4.48 (m), 4.44–4.35 (m), 4.35–4.23 (m), 4.19–4.06 (m), 2.49–2.28 (m), 2.31–2.15 (m), 2.08–1.70 (m), 1.69–1.55 (m), 1.54–1.43 (m), 1.45–1.20 (m). ^13^C NMR (101 MHz, CDCl_3_, CH_4_Si): δ 154.16, 84.70, 84.66, 84.51, 84.43, 82.25, 82.20, 82.02, 80.91, 80.72, 79.68, 79.55, 73.34, 73.28, 73.24, 73.06, 40.84, 40.78, 37.59, 37.58, 32.98, 32.88, 32.63, 32.55, 29.10, 29.00, 26.11, 26.10, 25.81, 25.67, 23.00, 22.91, 22.85, 22.46, 21.45, 21.06, 21.03, 20.98, 20.82, 20.66 ppm.

### General Procedure for the Continuous Conversion of Limonene Oxides under Optimized Conditions over 48 h

An empty HPLC column (250 mm × 4.6 mm × 1/4″ OD) was charged with **SILP 1** (2.22 g, loading: 30 wt % TBAC **1** for **7a**, 15 wt % TBAC **1** for **7b**), connected to the scCO_2_ device, and put in an oven, which was heated up to 120 °C. The back-pressure regulator was set to the appropriate pressure (15 MPa for **7a**, 20 MPa for **7b**). A 20 mL vial filled with substrate **7** was used as the substrate supply. The flow rates of the HPLC pumps were set to 0.01 mL/min (substrate **7a** and **7b**) and 1.99 mL/min (CO_2_, 2× 0.995 mL/min). The mixtures of the corresponding limonene oxide **7** and carbonate **8** were collected in 30 mL vials at different fractions. The collection time for each flask was set to 3 h, resulting in a total collection time of 48 h.

### Limonene Oxide **7a** as the Substrate for Continuous Conversion: Determination of NMR Yields

For the determination of NMR yields and conversions, naphthalene as the internal standard was added to each fraction (30 min fractions: 5.0 ± 0.1 mg (12 h experiments), 1 h fractions: 10.0 ± 0.1 mg (12 h experiments), 3 h: 30.0 ± 0.1 mg (48 h experiment), and 40.0 ± 0.1 mg (96 h experiment)) and homogenized with 0.5 mL of CDCl_3_. NMR measurements were performed with a 5–10 mg aliquot of the resulting mixtures. For the reference NMR spectrum (*t* = 0, see ESI Figure S12), 558 mg of limonene oxide **7a** (0.6 mL per 1 h, ρ = 0.93 g/mL) and 10 mg of naphthalene were mixed together.

### Limonene Dioxide **7b** as the Substrate for Continuous Conversion: Determination of GC Yields

For the determination of GC yields, the 3 h fractions were homogenized with 15 mL of ethyl acetate (ρ (**7b**) = 1.03 mg/mL; 123 mg of **7b**/mL of ethyl acetate, 12 h experiments: 5 mL of ethyl acetate). An aliquot of 12 μL of crude solution (30 min fractions: 24 μL (12 h experiments)), 30 μL of internal standard (20 mg octane/mL ethyl acetate), and 1458 μL (30 min fractions: 1446 μL (12 h experiments)) of ethyl acetate resulted in 1.5 mL of GC sample. The identity of the peaks was verified via GC/MS.

## Supporting Information Available

Synthesis and analysis of ionic liquids; characterization of SILPs via TGA, DRIFTS, and N_2_ physisorption (surface area: BET theory; porosity: BJH method); recycling studies of SILPs in batch mode; further studies and optimization works conducted in batch mode and under continuous flow; details about determination of NMR and GC yields; and analysis of products via NMR and GC (PDF)

## Abbreviations

[C_2_mim]X: 1-ethyl-3-methyl imidazolium halide
BET: Brunauer–Emmett–Teller (surface area)
BJH: Barret–Joyner–Halenda (pore size distribution)
DRIFTS: diffuse reflectance infrared Fourier transform spectroscopy
GC: gas chromatography
GC/MS: gas chromatography mass spectroscopy hyphenation
HPLC: high-performance liquid chromatography
NMR: nuclear magnetic resonance (spectroscopy)
scCO_2_: supercritical CO_2_
SILP: supported ionic liquid phase
TBAX: tetrabutylammonium halide
TGA: thermogravimetric analysis
UV: ultraviolet
