# Supplementary material for: Continuous Formation of Limonene Carbonates in Supercritical Carbon Dioxide
Source: Org Process Res Dev. 2022 Sep 16;26(10):2799–810. doi: 10.1021/acs.oprd.2c00143 (PMC9594339; doi:10.1021/acs.oprd.2c00143)
Supplement: Supplementary file 1 — op2c00143_si_001.pdf [file op2c00143_si_001.pdf]

Supporting Information

Continuous Formation of Limonene Carbonates in Supercritical Carbon Dioxide

Philipp Mikšovsky [1], Elias N. Horn [1], Shaghayegh Naghdi [2], Dominik Eder [2], Michael Schnürch [1] and Katharina Bica-Schröder [1,]*

[1]Institute of Applied Synthetic Chemistry (E163), TU Wien, Getreidemarkt 9/E163, 1060 Vienna, Austria.

[2]Institute of Materials Chemistry (E165), TU Wien, Getreidemarkt 9/E165, 1060 Vienna, Austria.

* Corresponding author: Katharina Bica-Schröder,

email: katharina.schroeder@tuwien.ac.at

phone: +43 1 58801 163601

Table of Contents

1 Materials and Methods 3

2 Ionic Liquids 4

2.1 Synthesis of [C_2mim]Br 5 and [C_2mim]I 6 4

2.2 NMR Spectra: 1-Ethyl-3-methylimidazolium Bromide [C_2mim]Br 5.......................................5

2.3 NMR Spectra: 1-Ethyl-3-methylimidazolium Iodide [C_2mim]I 6...6

3 Supported Ionic Liquid Phases (SILPs) 7

3.1 Thermogravimetric Analysis (TGA)..7

3.2 Diffuse Reflectance Infrared Fourier Transform Spectra (DRIFTS)..9

3.3 Nitrogen Physisorption Measurements (BET, BJH) 11

4 Conversion of Bioderived Limonene Oxide 7a..12

4.1 Determination of NMR Conversions and NMR Yields..12

4.2 Batch Conditions: Optimization Studies with SILP 1 as Catalyst 14

4.3 Batch Conditions: Recycling Studies of SILP 1 and SILP 2...15

4.4 Continuous Flow: Optimization of Flow Rates 15

4.5 Continuous Flow: Long-term Stability (96 h)...17

4.6 NMR Spectra: Limonene Carbonate 8a (Mixture of *cis* and *trans* Isomer) 18

5 Conversion of Bioderived Limonene Dioxide 7b..19

5.1 Determination of GC Yields: Calibration Curves and GC Chromatogram..............................19

5.2 Continuous Flow: Long-term Stability (48 h)..21

5.3 NMR and GC Spectra: Epoxycarbonate 8b (Mixture of 4 Diastereomers) 22

5.4 NMR and GC Spectra: Biscarbonate 8c (Mixture of 2 Diasteriomers)..................................24

6 List of Abbreviations...26

1 Materials and Methods

Detailed information about the $scCO_2$ flow device is summarized in the experimental part of the main manuscript.

Unless otherwise noted, chemicals were purchased from several chemical suppliers and used without further purification. Limonene dioxide **7b** was kindly provided by Nitrochemie Aschau GmbH. 1-Methylimidazole was distilled prior to use (80 °C, 13 mbar). Dichloromethane, used for reactions which required anhydrous conditions, were pre-distilled and dried over Al_2O_3 columns (PURESOLV, Innovative Technology). TLC analysis was performed on silica gel 60 F_{254} aluminium plates from Merck containing a fluorescent indicator using solvent mixtures of ethyl acetate in liquid petroleum. Spots were visualized using ultraviolet light (254 nm) or were stained with cer ammonium molybdate (0.5 g $Ce(NH_4)_4(SO_4)_4 \cdot 2H_2O$, 12.0 g $(NH_4)_6Mo_7O_{24} \cdot 4 H_2O$, 235 mL H_2O, 15 mL conc. H_2SO_4) followed by heating.

GC measurements were performed with a Thermo Scientific Trace 1310 gas chromatograph containing two capillary columns from Restek Rtx-5 (Rtx-5, 15 m x 0.25 mm x 1.00 µm) and a flame ionization detector (FID). A Thermo Trace 1300 / ISQ LT (single quadrupole MS (EI)) containing a standard capillary column from Restek (Rxi-5sil MS, 30 m x 0,25 mm x 0,25 µm) was used for GC/MS measurements (initial temperature: 100 °C (holding time 2 min); rate: 35 °C/min to 300 °C (holding time: 4 min).

^{1}H-NMR and ^{13}C-NMR spectra were recorded from $CDCl_3$ solutions on a Bruker Avance UltraShield *400/600* (^{1}H: 400, 600 MHz, ^{13}C: 101 MHz) spectrometer.

FTIR spectra (transmission mode) were recorded on a PerkinElmer Spectrum 65 FTIR spectrometer. Resolution was set to 4 cm^{-1}, 8 scans were used. Spectra were recorded from 4000 - 500 cm^{-1}. Raw data were processed with the PerkinElmer Spectrum Software.

DRIFT spectra (result spectra: Kubelka Munk) were recorded on a Bruker Vertex 80 FTIR spectrometer using a narrow band MCT detector. Resolution was set to 4 cm^{-1}, 256 scans were used. Spectra were recorded from 4000 – 800 cm^{-1} and raw data were processed with MestreNova and OPUS. Samples (40 mg / 400 mg KBr) were diluted with KBr from Sigma Aldrich (99%, FTIR grade) and dried for 4 days under high vacuum.

The physisorption measurements were carried out on an ASAP 2010 by Micromeritics GmbH. The degassing procedure was set to 120 °C for 6 hours and the isotherms were obtained using nitrogen at 77 K. BET (Brunauer-Emmett-Teller) specific surface areas were calculated based on BET equation, pore volumes were calculated based on BJH (Barret-Joyner-Halenda) equation from the desorption branch and average pore diameters were calculated based on desorption branch.

For thermogravimetric analysis (TGA), a Netzsch STA 449 F1 system was used. Temperature was increased from 25 °C to 450 °C with a rate of 5 °C/min.

2 Ionic Liquids

2.1 Synthesis of [C_2mim]Br 5 and [C_2mim]I 6

Tetrabutylammonium halides TBAX **1-3** and 1-ethyl-3-methylimidazolium chloride [C_2mim]Cl **4** were purchased from several suppliers and dried under high vacuum prior to use.

1-Ethyl-3-methylimidazolium bromide [C_2mim]Br **5** and 1-ethyl-3-methylimidazolium iodide [C_2mim]I **6** were synthesized according to modified literature protocols[1, 2]

Bromoethane or iodoethane (134 mmol, 1.10 equiv.) was added dropwise to freshly distilled 1-methylimidazole (10.00 g, 122 mmol, 1.00 equiv.) under argon atmosphere. For [C_2mim]Br **5**, the reaction mixture was heated up to 40 °C for 4.5 h, respectively to 80 °C for 30 min for [C_2mim]I **6**. Complete conversion was confirmed via ^{1}H-NMR spectroscopy. The reaction mixture was cooled with an ice bath whereby the product precipitated. The solid material was recrystallized (minimum amount of ACN for dissolving, ethyl acetate for precipitation). The solid material was washed with ethyl acetate (3 x 30 mL), volatiles were removed in *vacuo*. The ionic liquids **5** and **6** were dried for three days at room temperature under high vacuum. [C_2mim]Br **5**: 96% (22.35 g, 117 mmol, colorless crystals) ^{1}H NMR (400 MHz, $CDCl_3$, CH_4Si) δ = 10.28 (s, 1H, -N=C*H*-N-), 7.61 – 7.53 (m, 2H, -N-C*H*-C*H*-N-), 4.36 (q, *J* = 7.4 Hz, 2H, -N-CH_2-CH_3), 4.06 (s, 3H, -N-CH_3), 1.55 (t, *J* = 7.4 Hz, 3H, -N-CH_2-CH_3) ppm. ^{13}C NMR (101 MHz, $CDCl_3$, CH_4Si) δ = 137.04 (d, C2), 123.70 (d, C4), 121.97 (d, C5), 45.29 (t, *C*H_2-CH_3), 36.70 (q, N-*C*H_3), 15.71 (q, *C*H_2-*C*H_3) ppm. [C_2mim]I **6**: quant. (28.90 g, 121 mmol, pale yellow crystals) ^{1}H NMR (400 MHz, $CDCl_3$, CH_4Si) δ = 10.47 – 9.63 (m, 1H, -N=C*H*-N-), 7.50 (d, *J* = 1.7 Hz, 2H, -N-C*H*-C*H*-N-), 4.51 – 4.34 (m, 2H, -N-CH_2-CH_3), 4.11 (d, *J* = 0.6 Hz, 3H, -N-CH_3), 1.61 (t, *J* = 7.4 Hz, 3H, -N-CH_2-CH_3) ppm. ^{13}C NMR (101 MHz, $CDCl_3$, CH_4Si) δ = 136.76 (d, C2), 123.74 (d, C4), 121.99 (d, C5), 45.61 (t, *C*H_2-CH_3), 37.22 (q, N-CH_3), 15.75 (q, CH_2-*C*H_3) ppm.

2.2 NMR Spectra: 1-Ethyl-3-methylimidazolium Bromide [C_2mim]Br 5

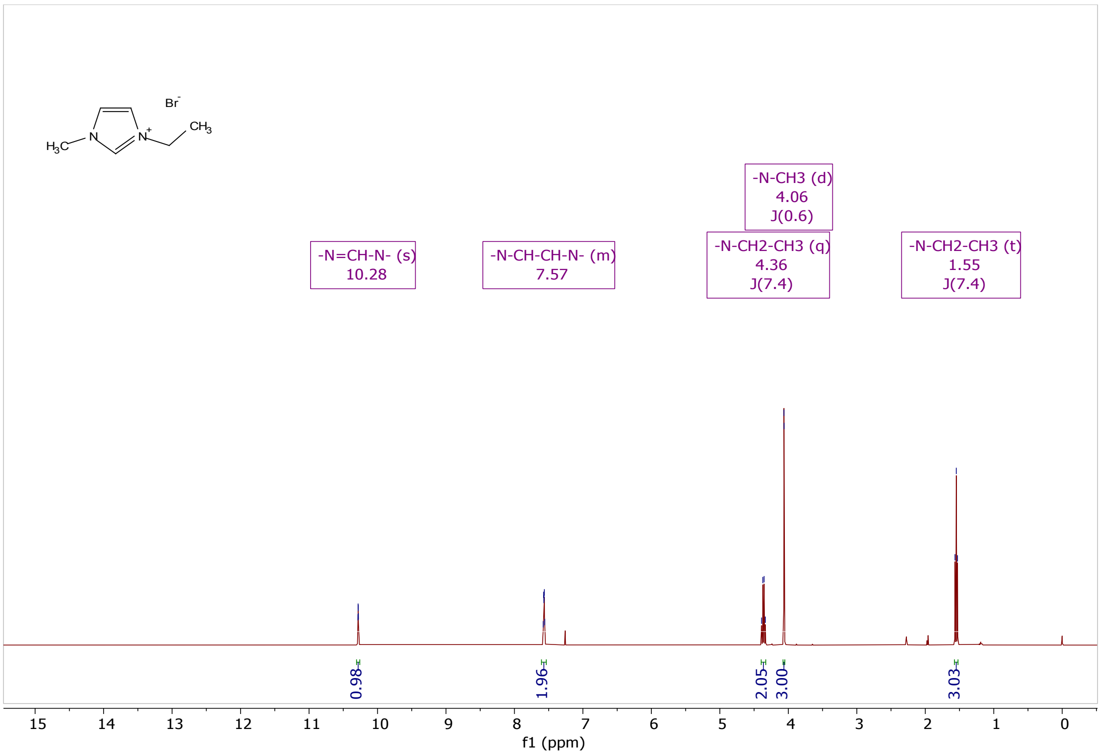

Figure S1: ^{1}H-NMR spectrum of 1-ethyl-3-methylimidazolium bromide [C_2mim]Br 5.

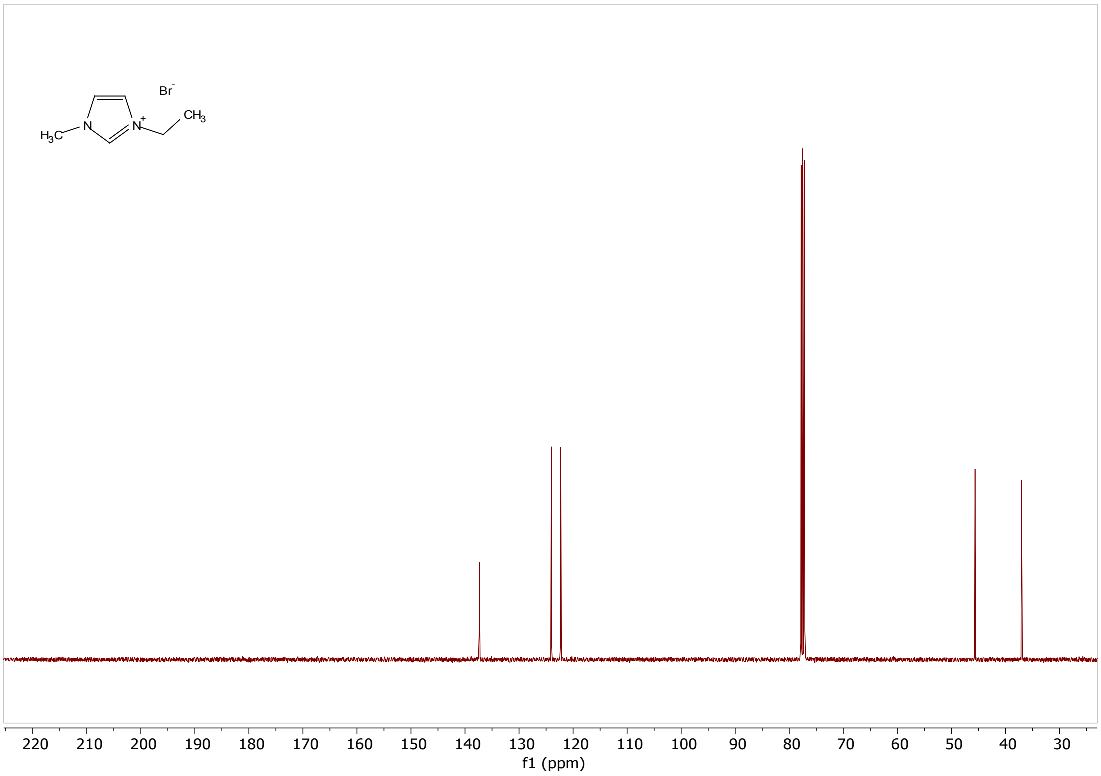

Figure S2: ^{13}C-NMR spectrum of 1-ethyl-3-methylimidazolium bromide [C_2mim]Br 5.

Spectral data are in accordance with literature[3].

2.3 NMR Spectra: 1-Ethyl-3-methylimidazolium Iodide [C_2mim]I 6

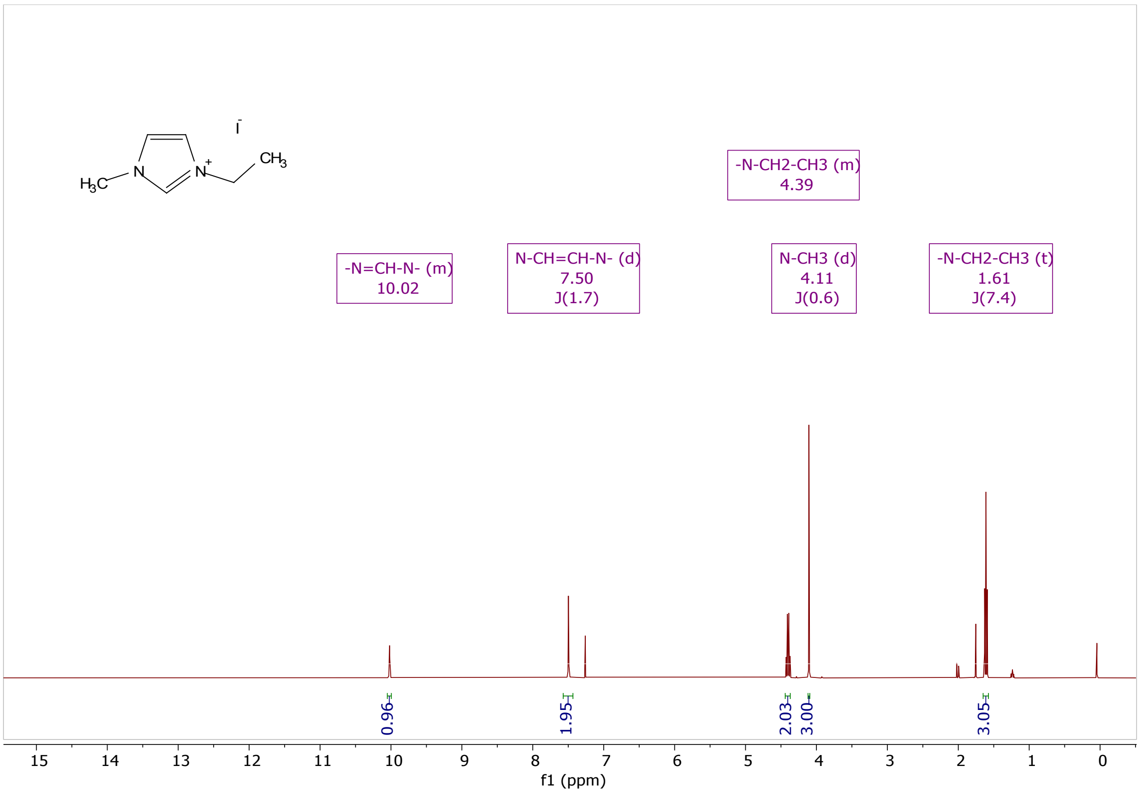

Figure S3: ^{1}H-NMR spectrum of 1-ethyl-3-methylimidazolium iodide [C_2mim]I 6.

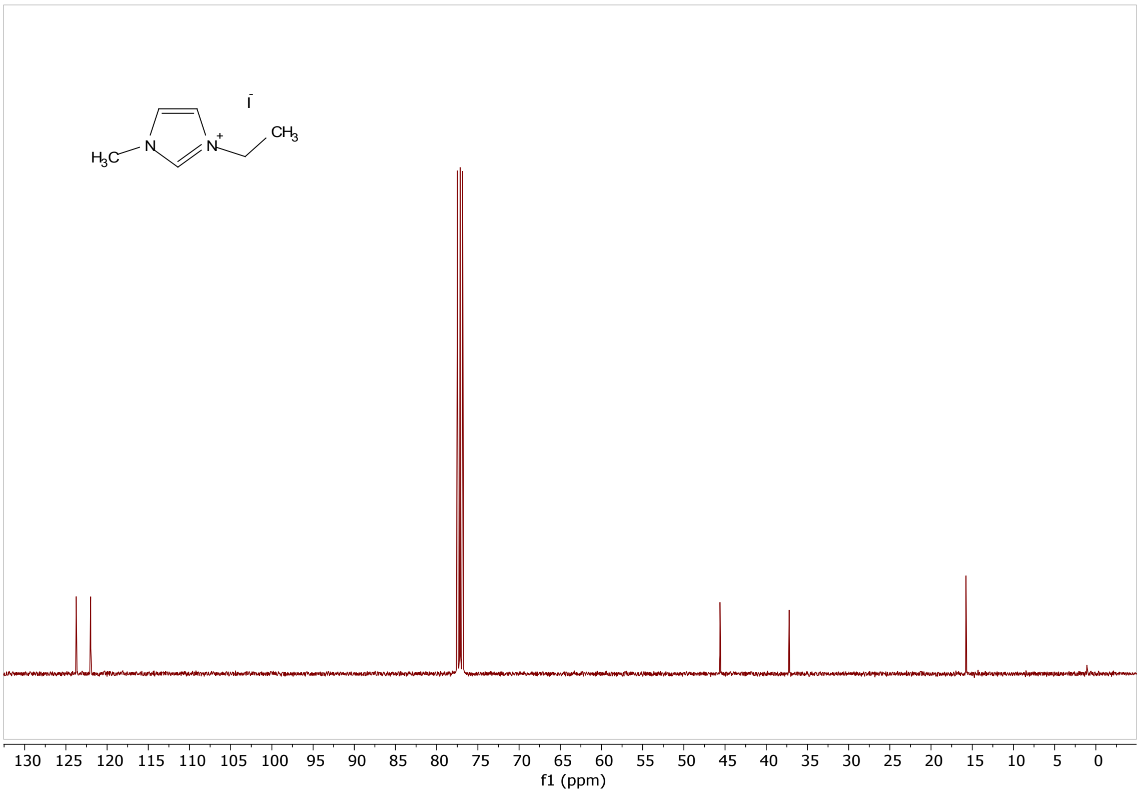

Figure S4: ^{13}C-NMR spectrum of 1-ethyl-3-methylimidazolium iodide [C_2mim]I 6.

Spectral data are in accordance with literature[4].

3 Supported Ionic Liquid Phases (SILPs)

3.1 Thermogravimetric Analysis (TGA)

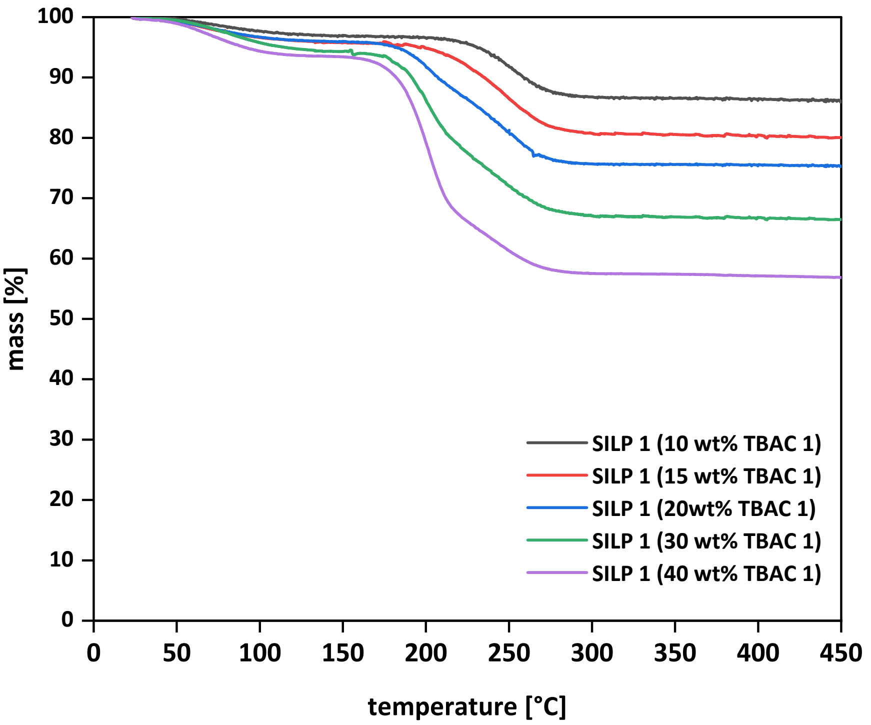

Figure S5: Catalyst loadings of SILP 1 measured via TGA.

Range: 25 °C – 450 °C (rate: 5 °C/min)

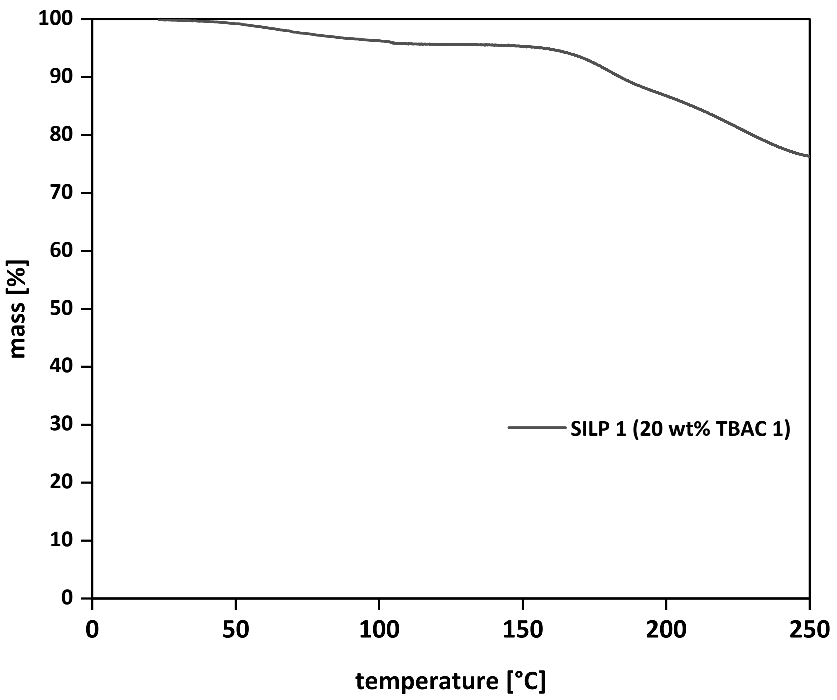

Figure S6: Thermal stability of SILP 1 (20 wt% TBAC 1) determined via TGA.

Range: 25 °C – 100 °C (rate: 5 °C/min); 100 °C – 250 °C (rate: 1 °C/min)

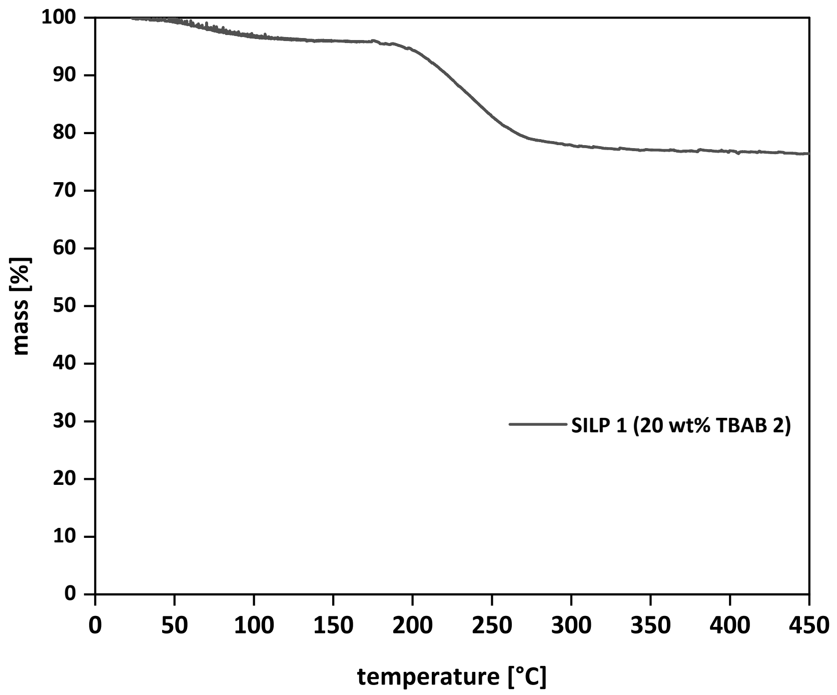

Figure S7: Catalyst loading of SILP 2 (20 wt% TBAB 2) measured via TGA.

Range: 25 °C – 500 °C (rate: 5 °C/min)

3.2 Diffuse Reflectance Infrared Fourier Transform Spectra (DRIFTS)

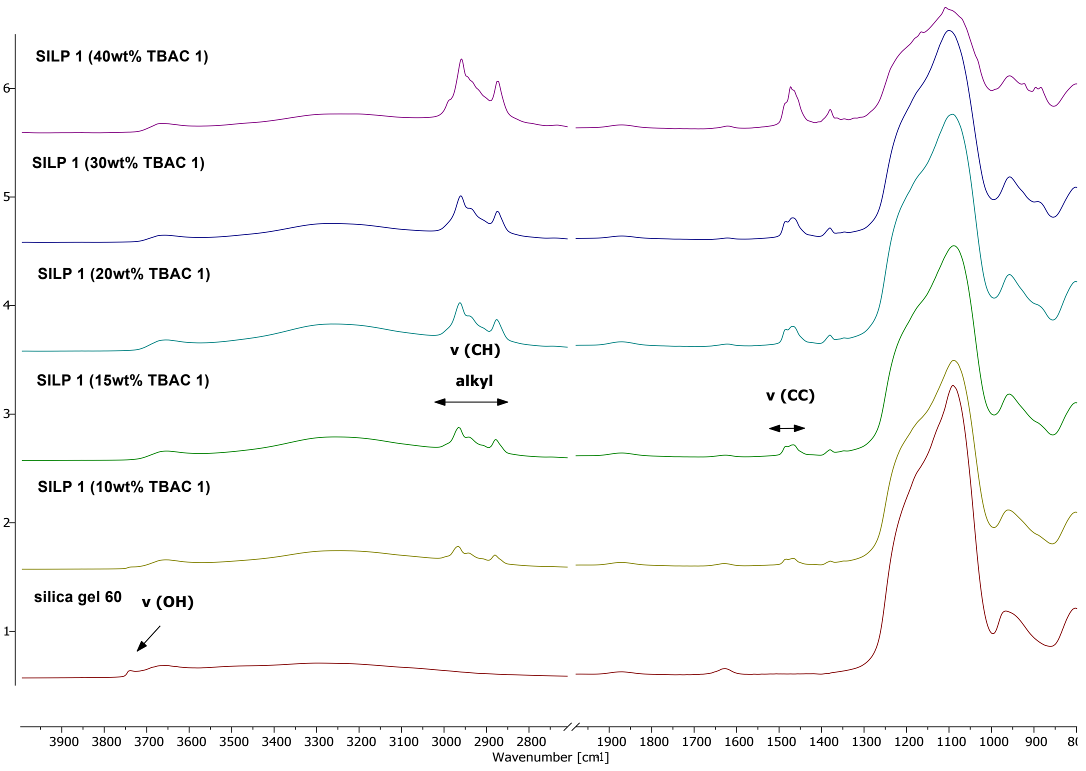

Figure S8: DRIFTS spectra of SILP 1 compared to supporting material silica gel 60

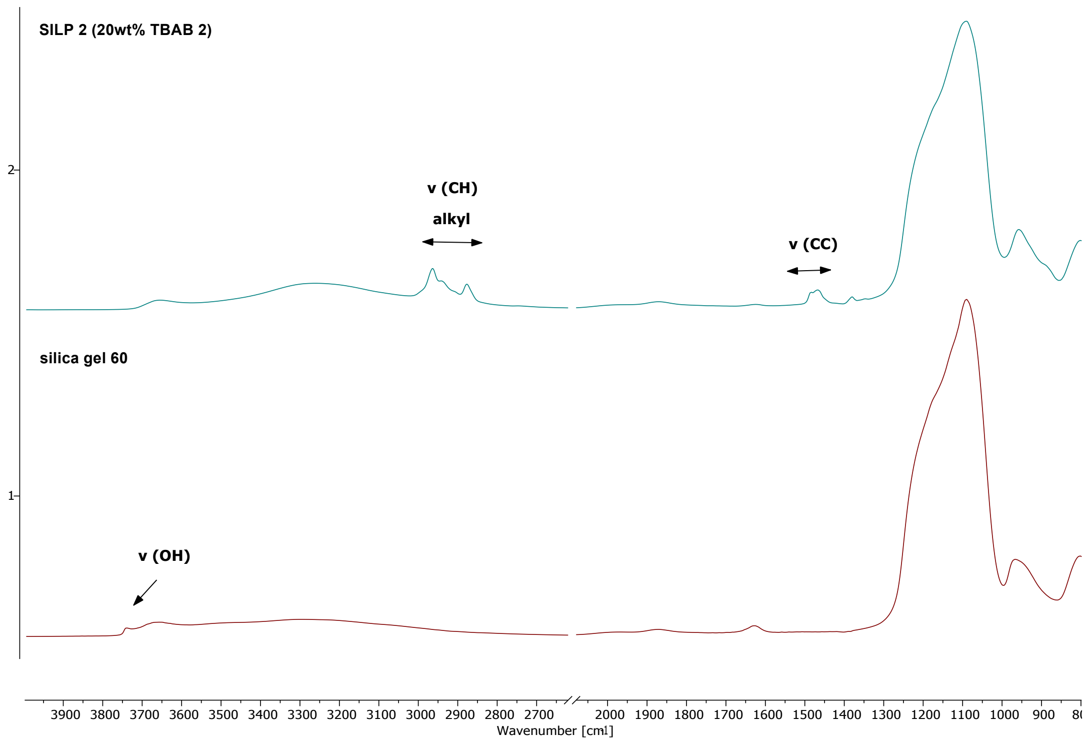

Figure S9: DRIFTS spectra of SILP 2 compared to supporting material silica gel 60

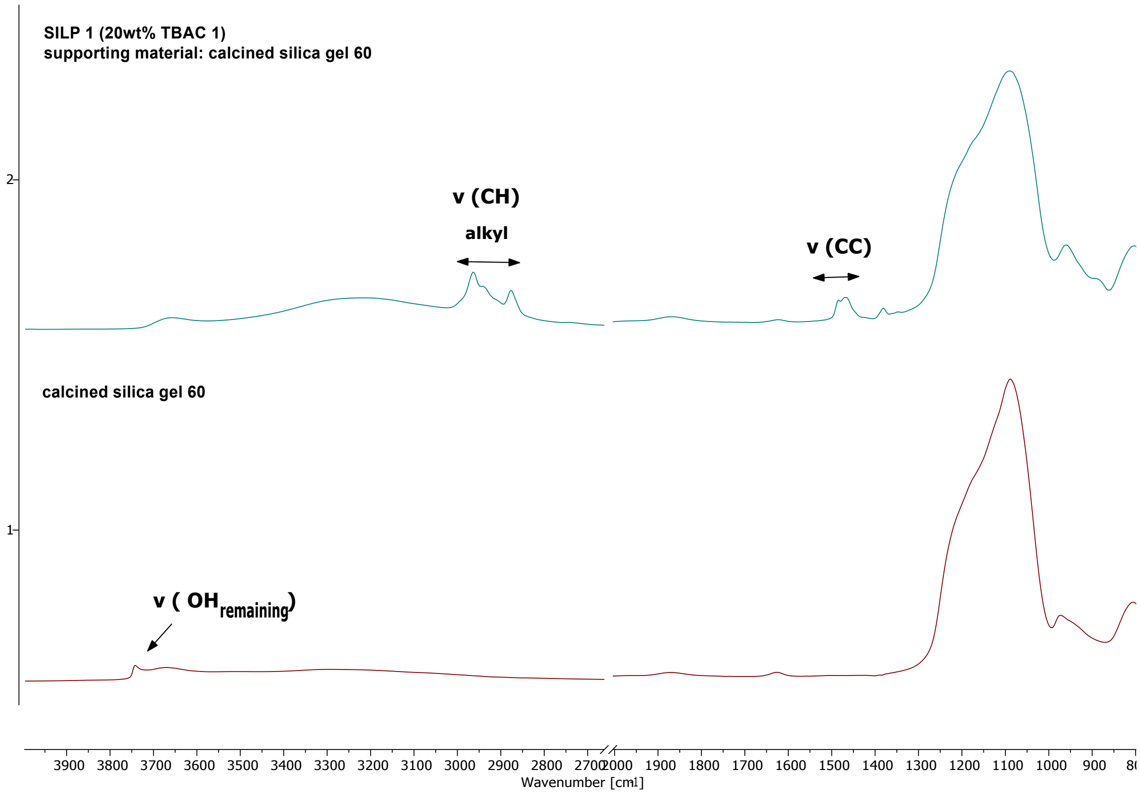

Figure S10: DRIFTS spectra of SILP 1 compared to supporting material calcined silica gel 60

3.3 Nitrogen Physisorption Measurements (BET, BJH)

Table S1: Characterization of SILP 1 (15 wt% catalyst loading) via N_2 physisorption

sample	BET surface area[a] [m²/g]	pore volume[b] [cm³/g]	average pore diameter[c] [Å]
silica gel 60 (reference)	634.37	0.91	55.38
**SILP 1** (15 wt% TBAC 1 - freshly prepared)	450.93	0.57	49.07
**SILP 1** (15 wt% TBAC 1 – after 48 h reaction time)	231.01	0.34	45.40

[a] calculated based on BET equation; [b] calculated based on BJH equation; determined based on desorption branch

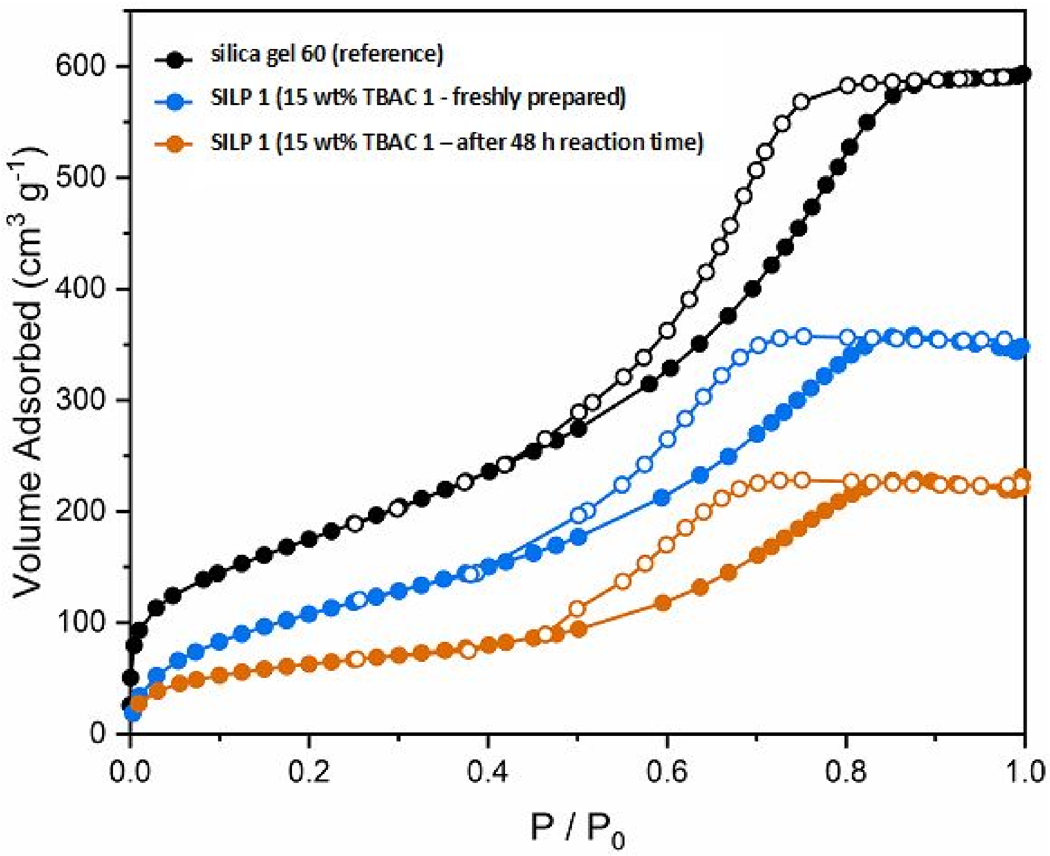

Figure S11: N_2 adsorption – desorption isotherms of silica gel 60 (reference material, black), freshly prepared SILP 1 catalyst (blue) and SILP 1 catalyst after 48 h reaction time (orange)

4 Conversion of Bioderived Limonene Oxide 7a

4.1 Determination of NMR Conversions and NMR Yields

On the Example of a Batch Reaction Running for 20 h

For the determination of yields and conversions, an NMR spectrum of the reaction mixture before (t=0) and after (t=20) the reaction was recorded (see Figure S11). The NMR conversions and NMR yields were determined according to formulas S1-S6 given below by comparison of the integrals of the same hydrogens of the starting material before the reaction and the product after the reaction. The integrals are always referenced to the same amount of internal standard (naphthalene, δ = 7.82 and 7.45 ppm, integral set to 1.00).

In case of the continuous flow experiments, no conversions were determined, incorrect values were determined due to partial evaporation of the volatile starting material while CO_2 was released via the back-pressure regulator.

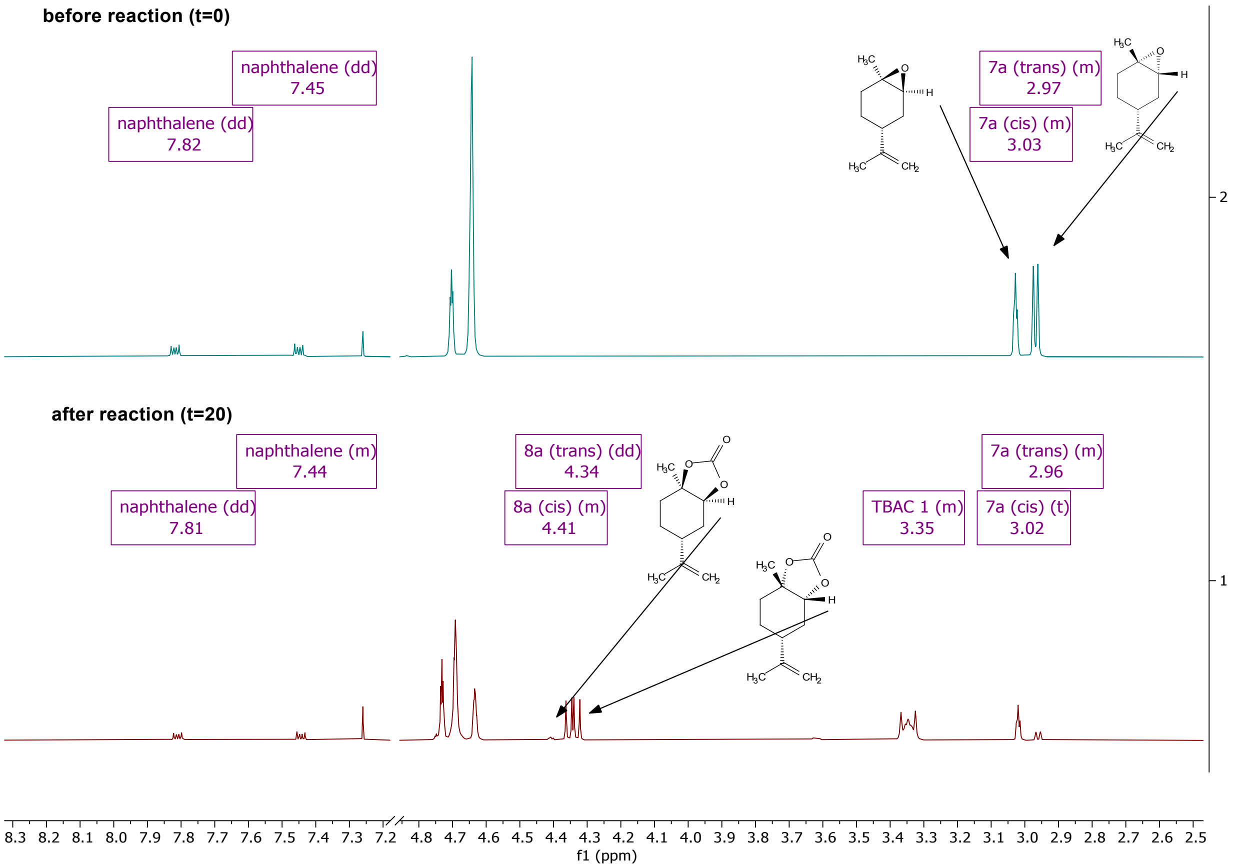

Figure S12: Limonene oxide 7a: Calculation of NMR yields via the comparison of the integrals of recorded ^{1}H-NMR spectra before and after the reaction.

$$\text{conversion } \mathbf{7a}\ (cis)\ (\%) = \frac{I_{7a\,(cis)_{t=0}} - I_{7a\,(cis)_{t=20}}}{I_{7a\,(cis)_{t=0}}} \cdot 100$$

Formula S1

$$\text{conversion } \mathbf{7a}\ (trans)\ (\%) = \frac{I_{7a\,(trans)_{t=0}} - I_{7a\,(trans)_{t=20}}}{I_{7a\,(trans)_{t=0}}} \cdot 100$$

Formula S2

$$\text{conversion } \mathbf{7a}\ (\%) = \frac{(I_{7a\,(cis)\,t=0} + I_{7a\,(trans)_{t=0}}) - (I_{7a\,(cis)\,t=20} + I_{7a\,(trans)_{t=20}})}{I_{7a\,(cis)_{t=0}} + I_{7a\,(trans)_{t=0}}} \cdot 100$$

Formula S3

$$\text{yield } \mathbf{8a}\ (cis)\ (\%) = \frac{I_{8a\,(\mathrm{cis})_{t=20}}}{I_{8a\,(\mathrm{cis})_{t=0}} + I_{8a\,(\mathrm{cis})_{t=0}}} \cdot 100$$

Formula S4

$$\text{yield } \mathbf{8a}\ (trans)\ (\%) = \frac{I_{8a\,(trans)_{t=20}}}{I_{7a\,(cis)_{t=0}} + I_{7a\,(trans)_{t=0}}} \cdot 100$$

Formula S5

$$\text{yield } \mathbf{8a}\ (\%) = \frac{I_{8a\,(cis)_{t=20}} + I_{8a\,(trans)_{t=20}}}{I_{7a\,(cis)_{t=0}} + I_{7a\,(trans)_{t=0}}} \cdot 100$$

Formula S6

conversion (**7a**)	conversion of *cis* and *trans* limonene oxide **7a**
yield (**8a**)	yield of *cis* and *trans* limonene carbonate **8a**
$I_{7a\,(\mathrm{xxx})_{t=yy}}$	integral of *cis* or *trans* limonene oxide **7a** at t= 0 h or 20 h
$I_{8a\,(\mathrm{xxx})_{t=20}}$	integral of *cis* or *trans* limonene carbonate **8a** after the reaction (t=20)

4.2 Batch Conditions: Optimization Studies with SILP 1 as Catalyst

Table S2: Limonene oxide 7a: Summary of optimization studies with SILP 1. [a]

entry	solvent	temperature	catalyst loading	supporting material [b]	yield of 8a (NMR) [%]
S1	neat	100 °C	20 wt%	silica	62
S2	+ 10 wt% H_2O				31 (+20% limonene diol[c])
S3	heptane				41
S4	neat	80 °C	20 wt%	silica	42
S5		100 °C			62
S6		120 °C			60
S7	neat	100 °C	10 wt%	silica	26
S8			15 wt%		30
S9			20 wt%		62
S10			40 wt%		68
S11	neat	100 °C	20 wt%	silica	62
S12				calcined silica	45

[a] conditions: 5 MPa CO_2 (gaseous, initial pressure), 5 mmol limonene oxide **7a** (*cis*/*trans*=43/57), 10 mol% TBAC **1** immobilized as **SILP 1**, 13 mg naphthalene (internal standard), 100 °C, 20 h; Further information about the calculations of NMR yields are summarized in the supplementary information (ESI Figure S12 and Formula S1-S6); [b] supporting materials were dried in a vacuum oven (50 mbar, 50 °C, 24 h) prior to use; calcination of silica was performed at 400 °C for 3 days; [c] according to GC/MS.

4.3 Batch Conditions: Recycling Studies of SILP 1 and SILP 2

Table S3: Limonene oxide 7a: Recycling of SILPs in batch mode

cycle	catalyst	yield (NMR) [%] [a] sum
cycle 1	**SILP 1** (20 wt% TBAC **1**)	62
cycle 2		50
cycle 3		30
cycle 4		25
cycle 1	**SILP 2** (20 wt% TBAB **2**)	31
cycle 2		31
cycle 3		29
cycle 4		23

[a] Conditions: 5 MPa CO_2 (gaseous, initial pressure), 5 mmol limonene oxide **7a** (*cis/trans*=43/57), 10 mol% catalyst **1-6**, 13 mg naphthalene (internal standard), 100 °C, 20 h; work-up: separation of SILP material via suck filtration, washing with heptane and evaporation of volatiles; Conversions were not determined due to partial removal of volatile starting material during evaporation of heptane. Further information about the calculations of NMR yields are summarized in the supplementary information (ESI Figure S12 and Formula S1-S6).

4.4 Continuous Flow: Optimization of Flow Rates

Table S4: Limonene oxide 7a: Influence of flow rates of CO_2 and substrate in continuous flow using SILP 1 as heterogeneous catalyst. [a]

entry	flow rates [mL/min]		residence time [s]	yield (NMR) [b] [%]		leaching [c]
	CO_2	substrate		maximum	overall (12 h)	
S13	0.99		250 s	44	19	< 1%
S14	1.49		166 s	28	18	< 1%
S15	1.99	0.01	125 s	22	15	n. o.
S16	2.49		100 s	22	16	n. o.
S17	3.99		62 s	17	12	n. o.
S18	1.98	0.02	125 s	20	14	< 1%

[a] Conditions: **SILP 1** (2.222 g, 250 mm column), 15 MPa, 120 °C, 12 h; [b] Yields are given as sum of *cis* and *trans* isomer. internal standard: naphthalene; Further information about the calculations of NMR yields are summarized in the supplementary information (ESI Figure S12 and Formula S1-S6); [c] for determination of leaching, the integral of the signal at δ = 3.35 ppm of TBAC **1** was used.

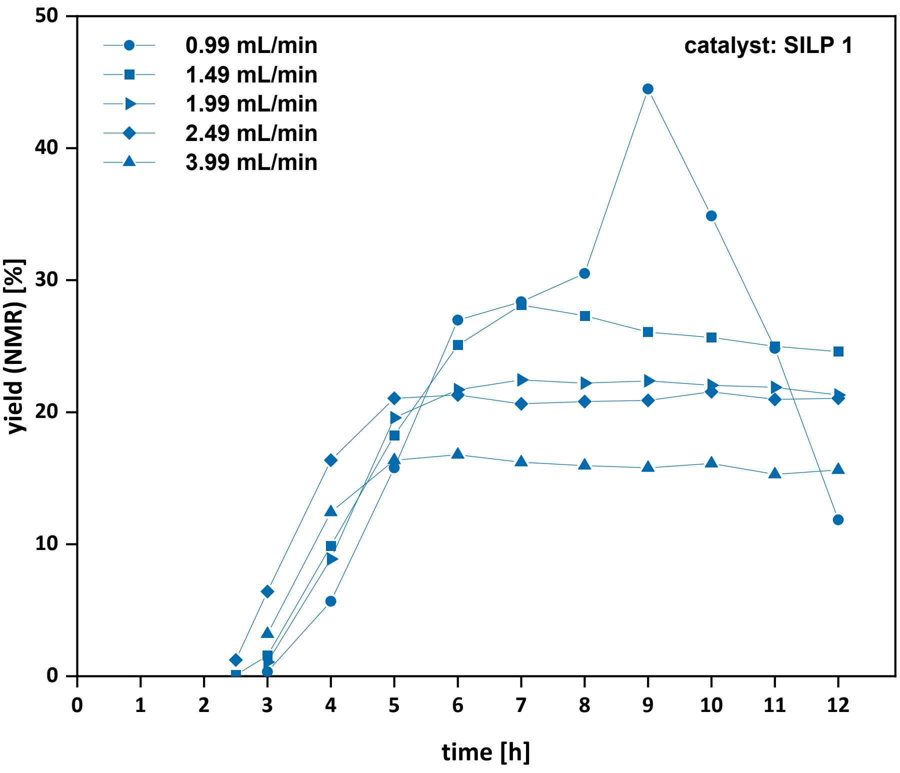

Figure S13: Limonene oxide 7a: Impact of flow rates of CO_2 in continuous flow.

4.5 Continuous Flow: Long-term Stability (96 h)

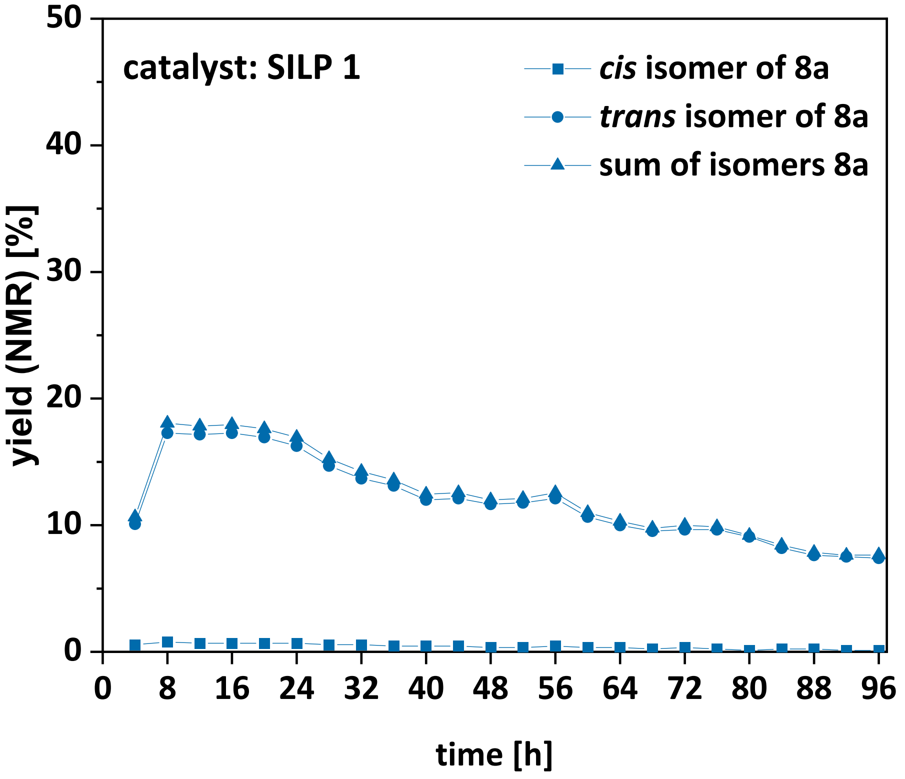

Figure S14: Limonene oxide 7a: Long-term stability of SILP 1 over 96 h. Final optimized conditions: SILP 1 (2.22 g, 30 wt% loading), 1.99 mL/min CO_2, 0.01 mL/min limonene oxide 7a, 15 MPa, 120 °C, 96 h, 250 mm catalyst cartridge

4.6 NMR Spectra: Limonene Carbonate 8a (Mixture of *cis* and *trans* Isomer)

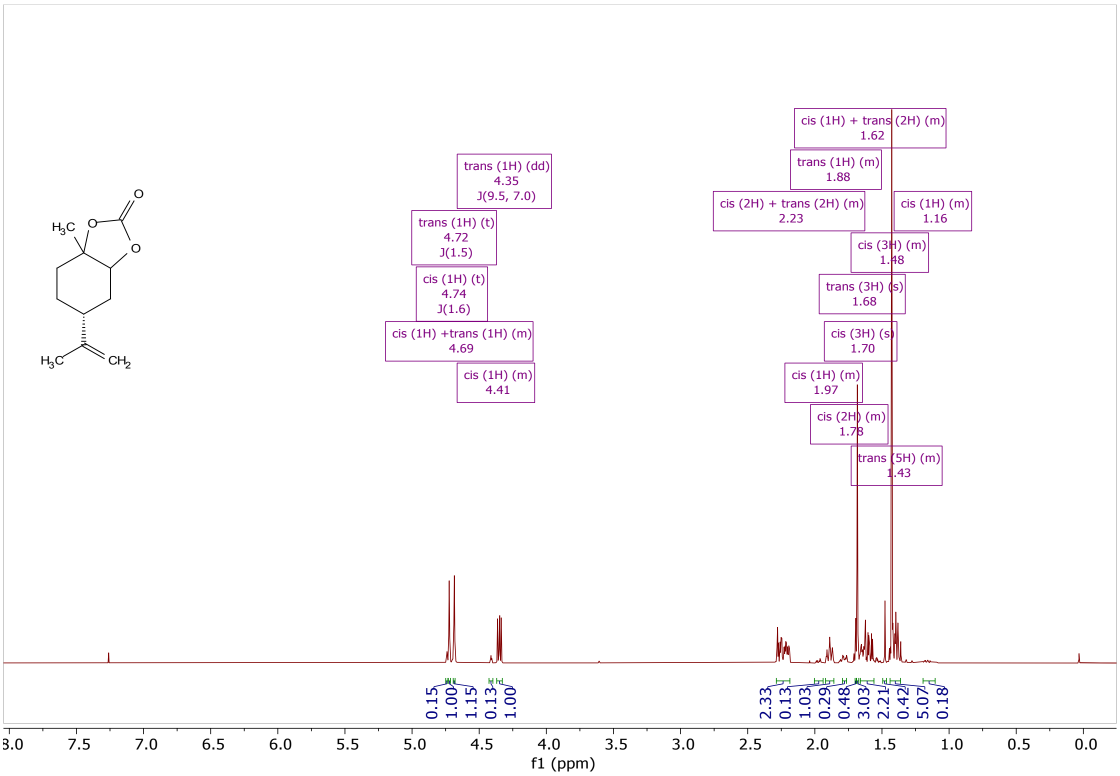

Figure S15: ^{1}H-NMR spectrum of the diastereomeric mixture of limonene carbonate 8a.

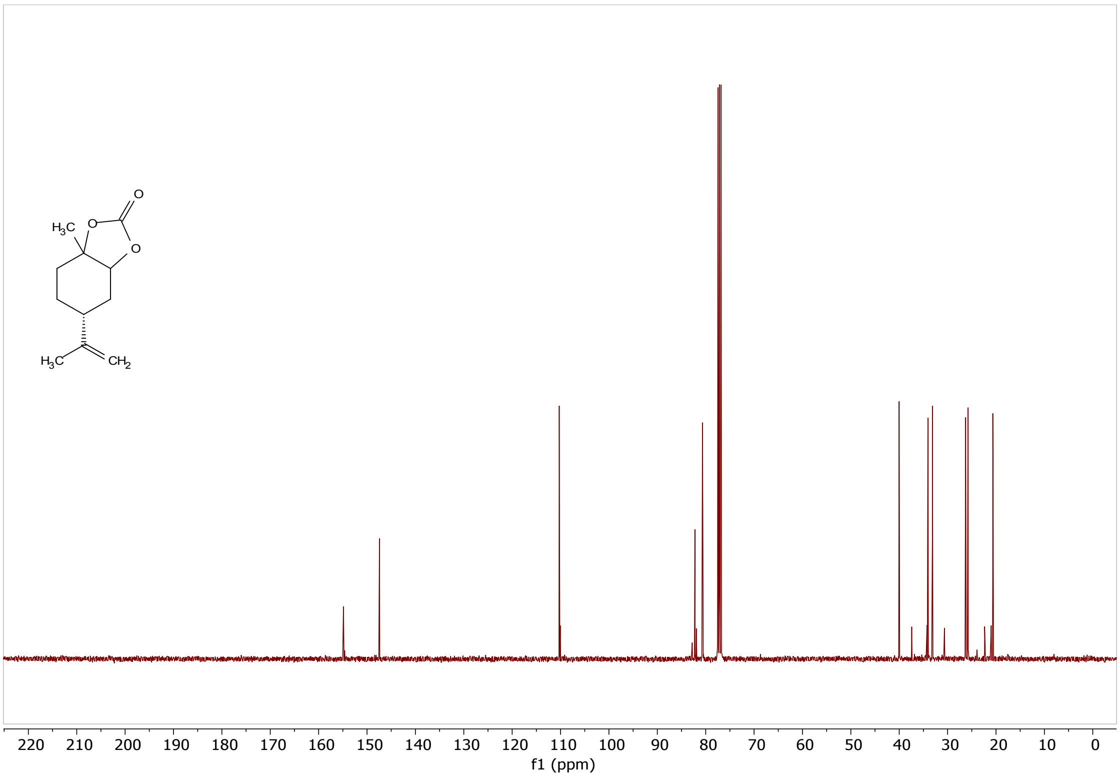

Figure S16: ^{13}C-NMR spectrum of the diastereomeric mixture of limonene carbonate 8a.

Spectral data are in accordance with literature.[5]

5 Conversion of Bioderived Limonene Dioxide 7b

5.1 Determination of GC Yields: Calibration Curves and GC Chromatogram

For the calibration curves, epoxycarbonate **8b** and biscarbonate **8c** were purified via column chromatography and a dilution series of each compound in ethyl acetate (1, 0.5, 0.25, 0.125, 0.0625 mg/mL) was prepared using octane as internal standard (0.4 mg/mL sample).

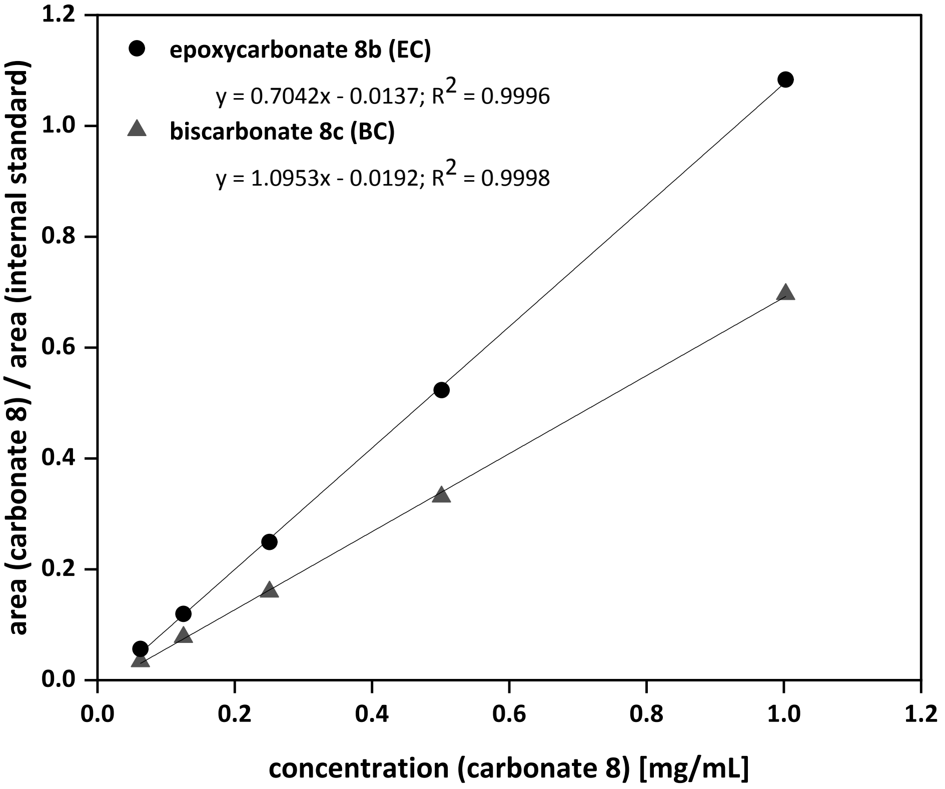

Figure S17: GC calibration curves for epoxycarbonate 8b and biscarbonate 8c.

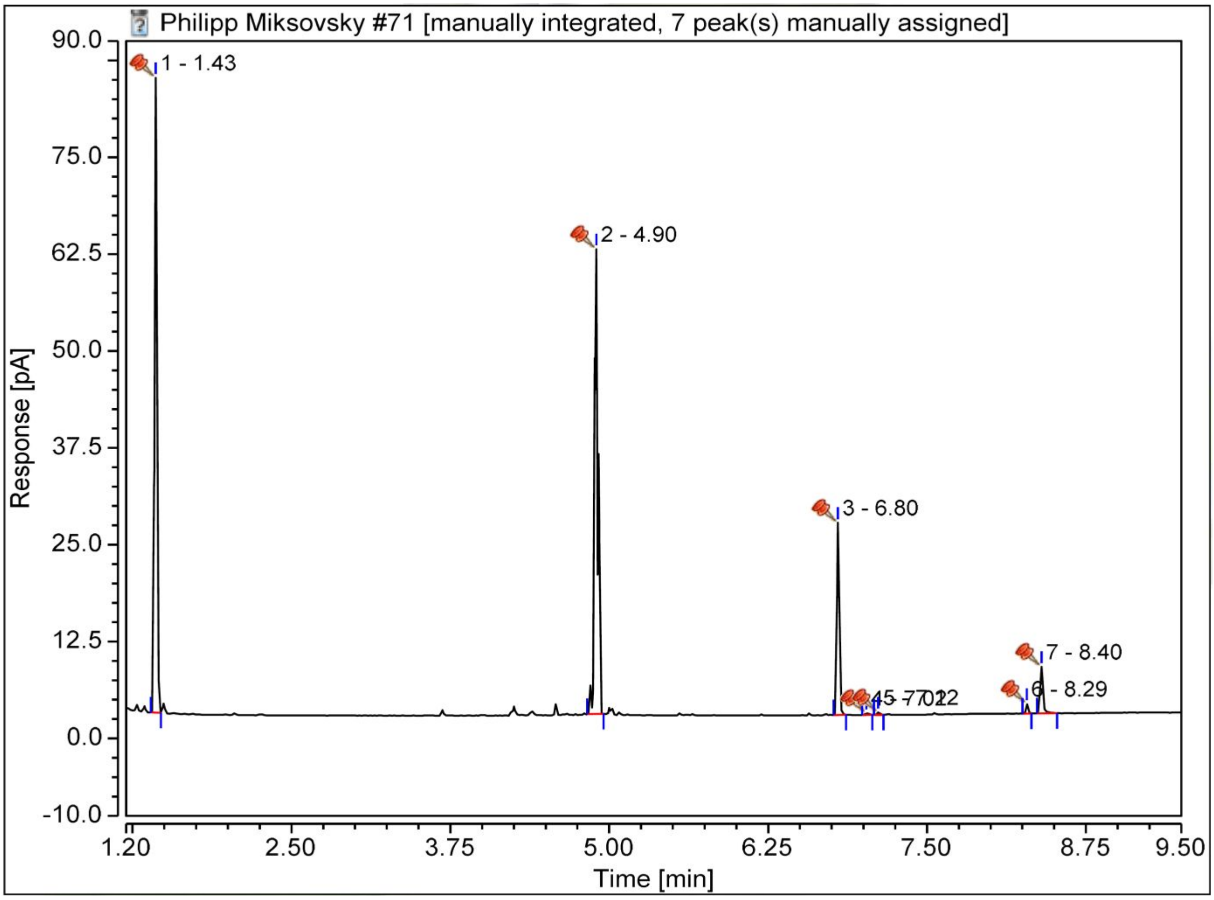

No.	Peak Name	Retention Time min	Area pA*min	Height pA	Relative Area %
1	internal standard (octane)	1.432	1.775	81.962	40.25
2	limonene dioxide	4.899	1.917	60.008	43.45
3	epoxy carbonate (EC1)	6.799	0.528	24.805	11.98
4	epoxy carbonate (EC2+3)	7.022	0.007	0.235	0.16
5	epoxy carbonate (EC4)	7.116	0.007	0.299	0.17
6	biscarbonate (BC1)	8.286	0.026	1.259	0.58
7	biscarbonate (BC2)	8.400	0.150	6.021	3.41
**Total:**			**4.410**	**174.589**	**100.00**

Figure S18: Limonene dioxide 7b: A typical gas chromatogram for the calculation of GC-yields.
The sum of isomers of epoxycarbonate **8b** (EC 1-4) and biscarbonate **8c** (BC 1-2) was used for calculations of GC yields.

5.2 Continuous Flow: Long-term Stability (48 h)

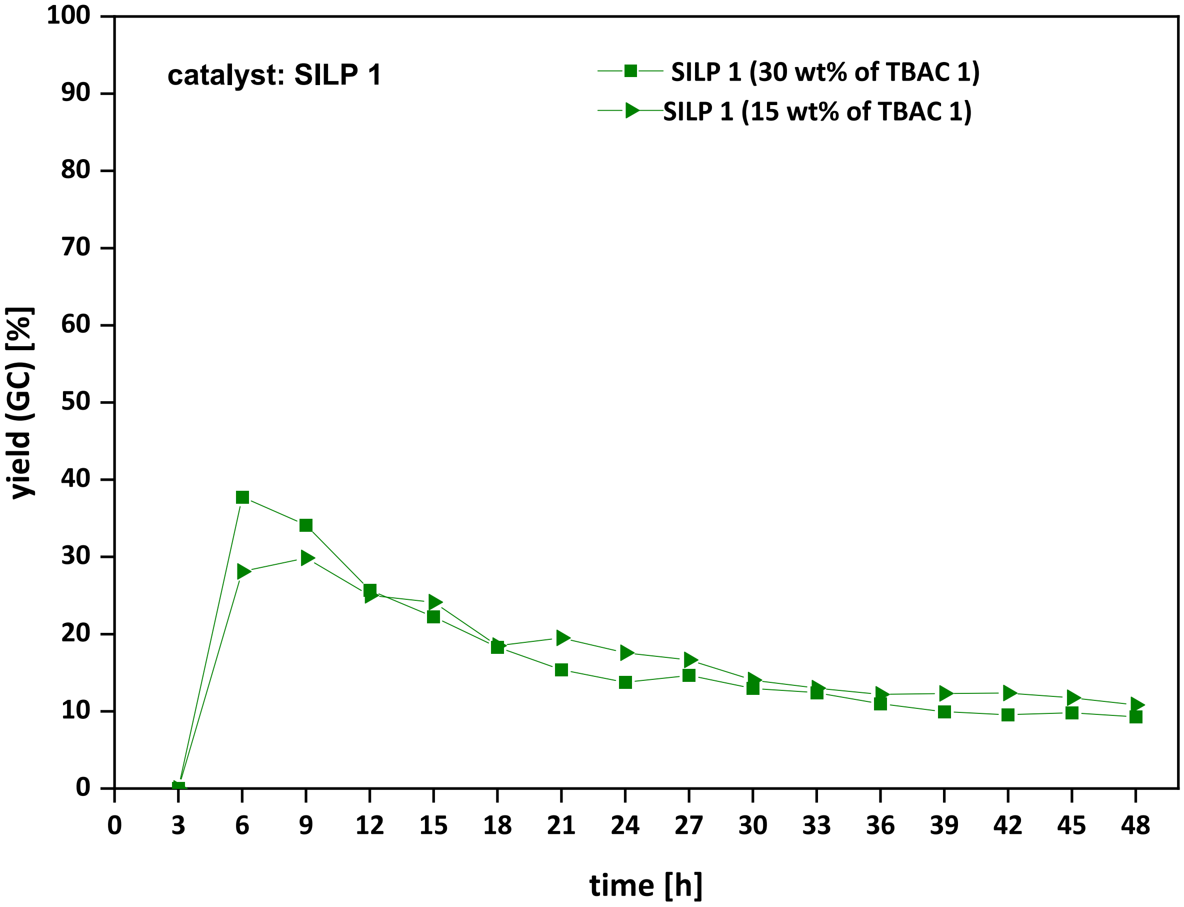

Figure S19: Limonene dioxide 7b: Long-term stability of SILP 1 using catalyst loadings of 15 wt% and 30 wt%. Conditions: SILP 1 (2.22 g), 1.99 mL/min CO2, 0.01 mL/min limonene dioxide 7b, 20 MPa, 120 °C, 48 h; leaching of immobilized catalyst TBAC 1 was quantified via 1H-NMR spectroscopy (signal at 3.35 ppm) using naphthalene as internal standard.

5.3 NMR and GC Spectra: Epoxycarbonate 8b (Mixture of 4 Diastereomers)

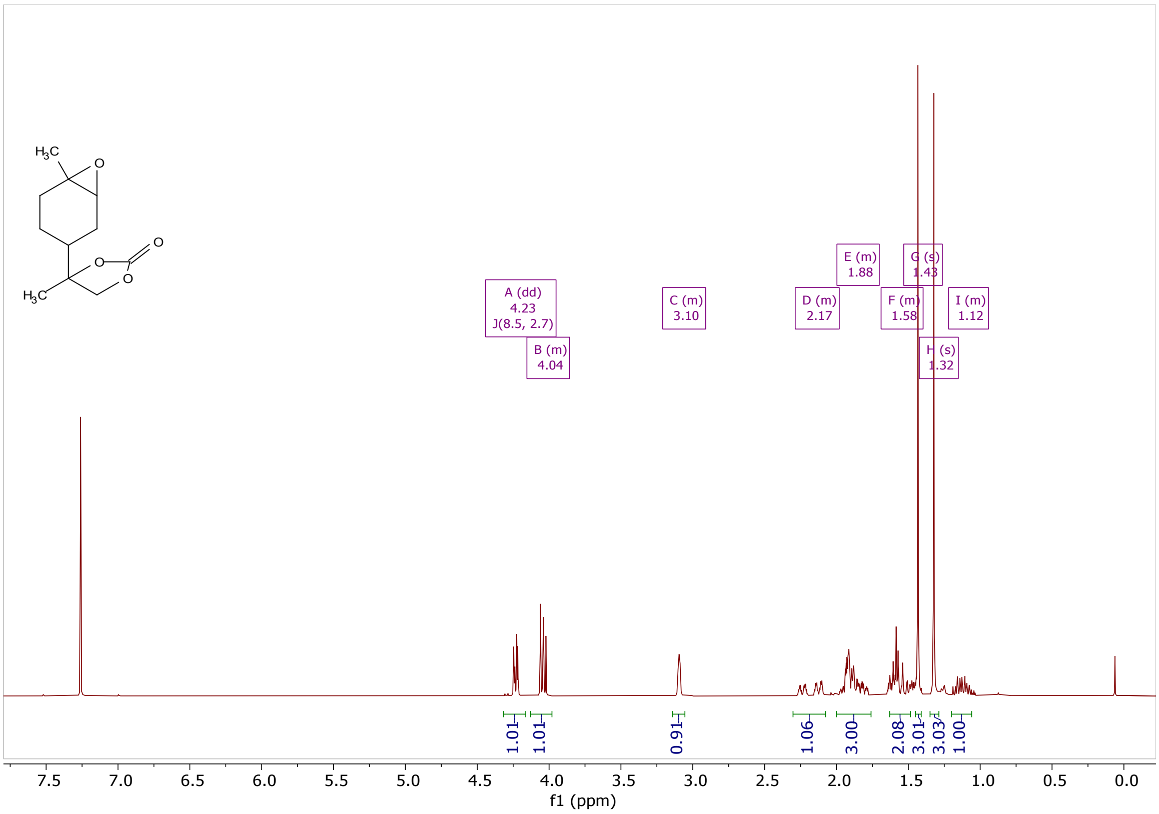

Figure S20: ^{1}H-NMR spectrum of the diastereomeric mixture of epoxycarbonate 8b.

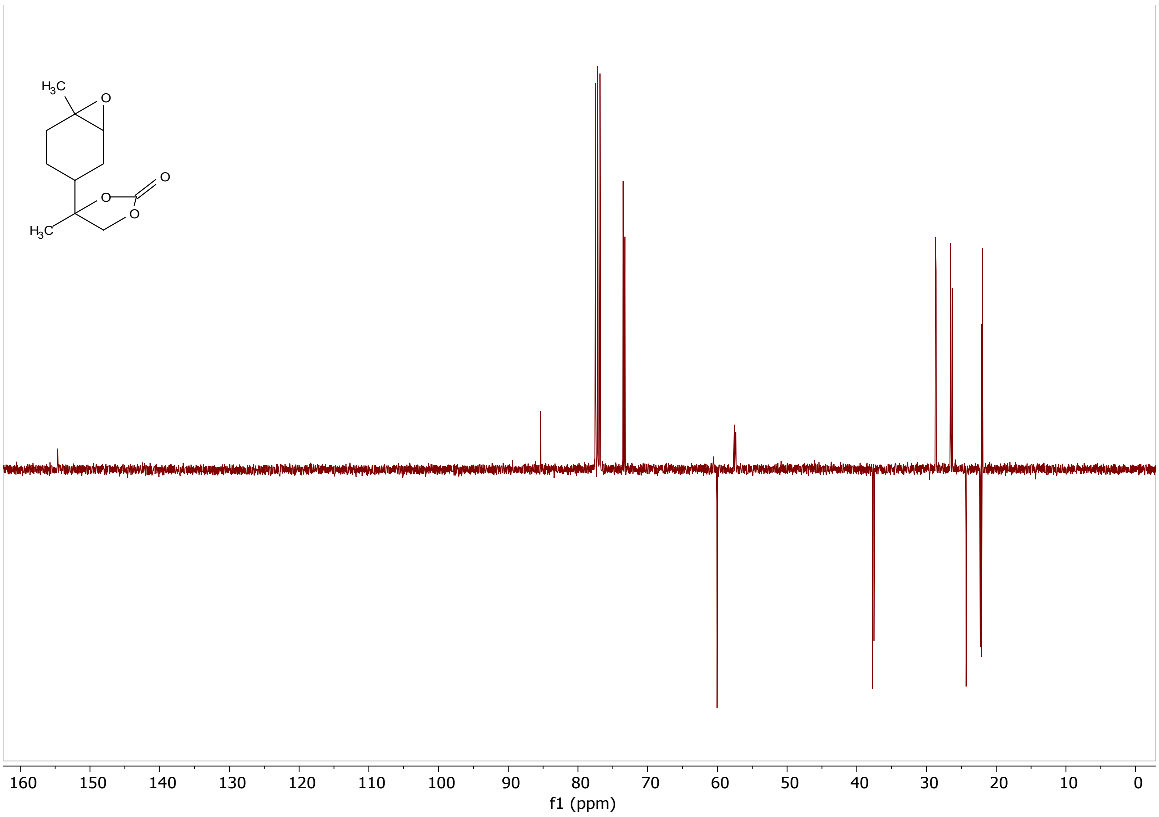

Figure S21: ^{13}C-NMR spectrum of the diastereomeric mixture of epoxycarbonate 8b.

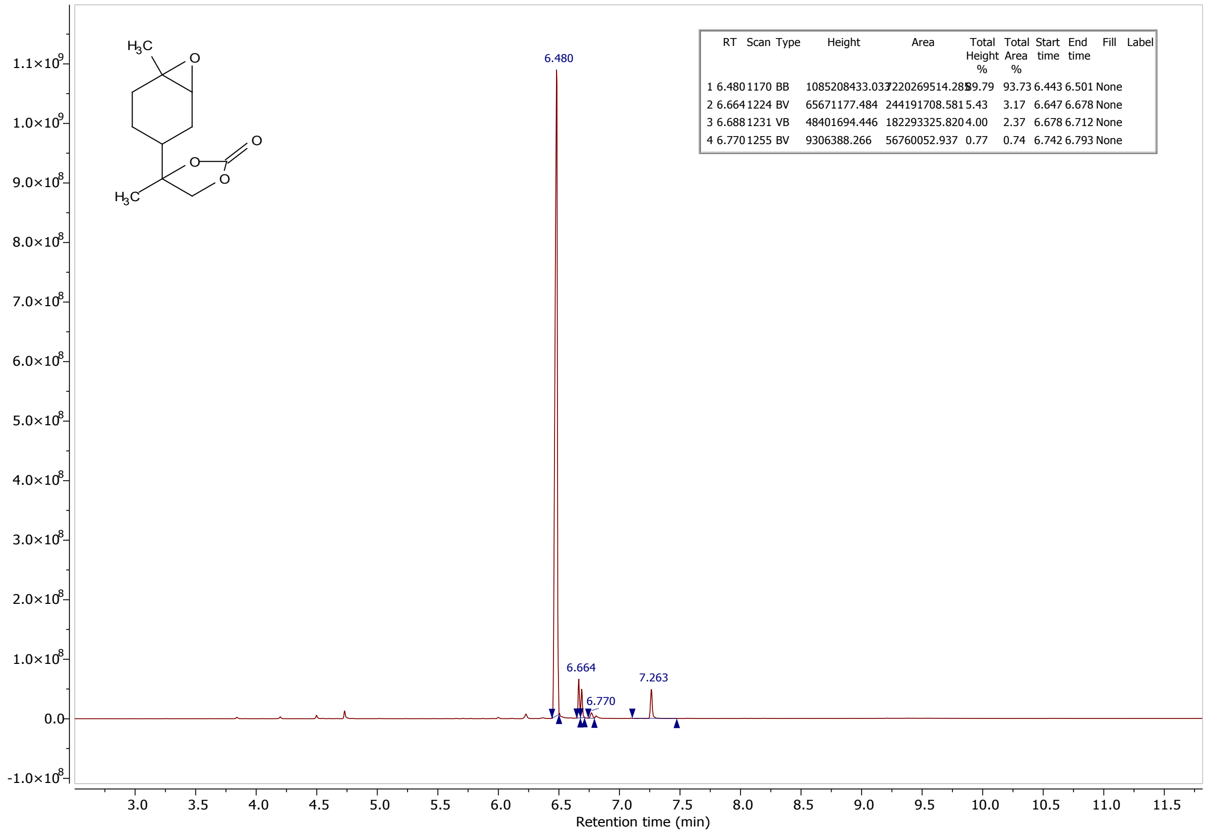

Figure S22: Gas chromatogram of the diastereomeric mixture of epoxycarbonate 8b.

5.4 NMR and GC Spectra: Biscarbonate 8c (Mixture of 2 Diasteriomers)

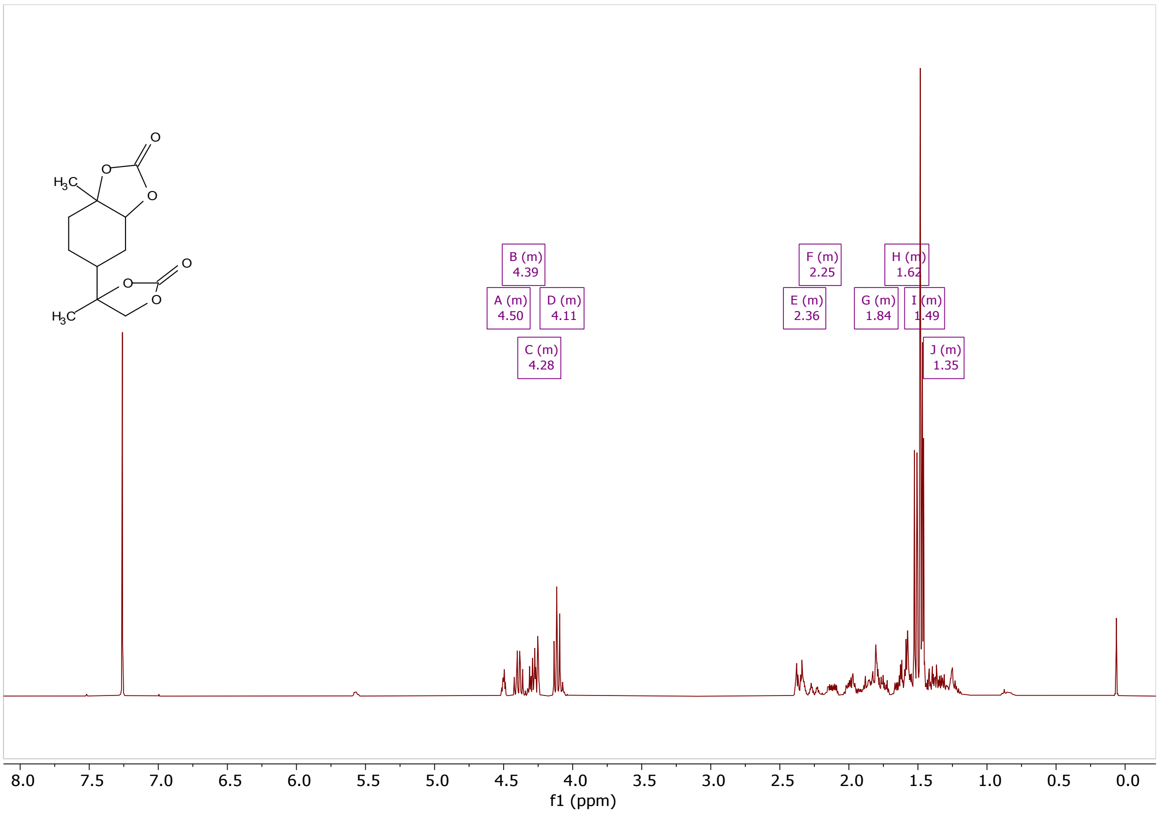

Figure S23: ^{1}H-NMR spectrum of the diastereomeric mixture of biscarbonate 8c.

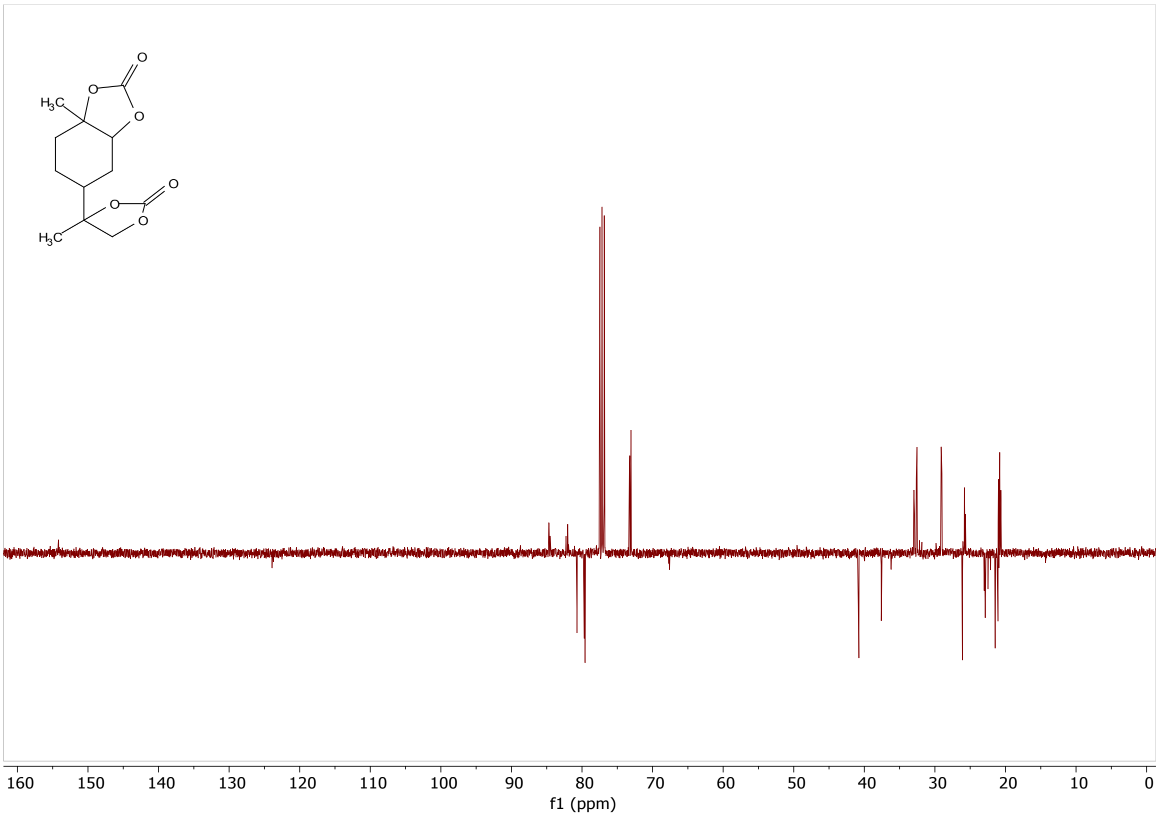

Figure S24: ^{13}C-NMR spectrum of the diastereomeric mixture of biscarbonate 8c.

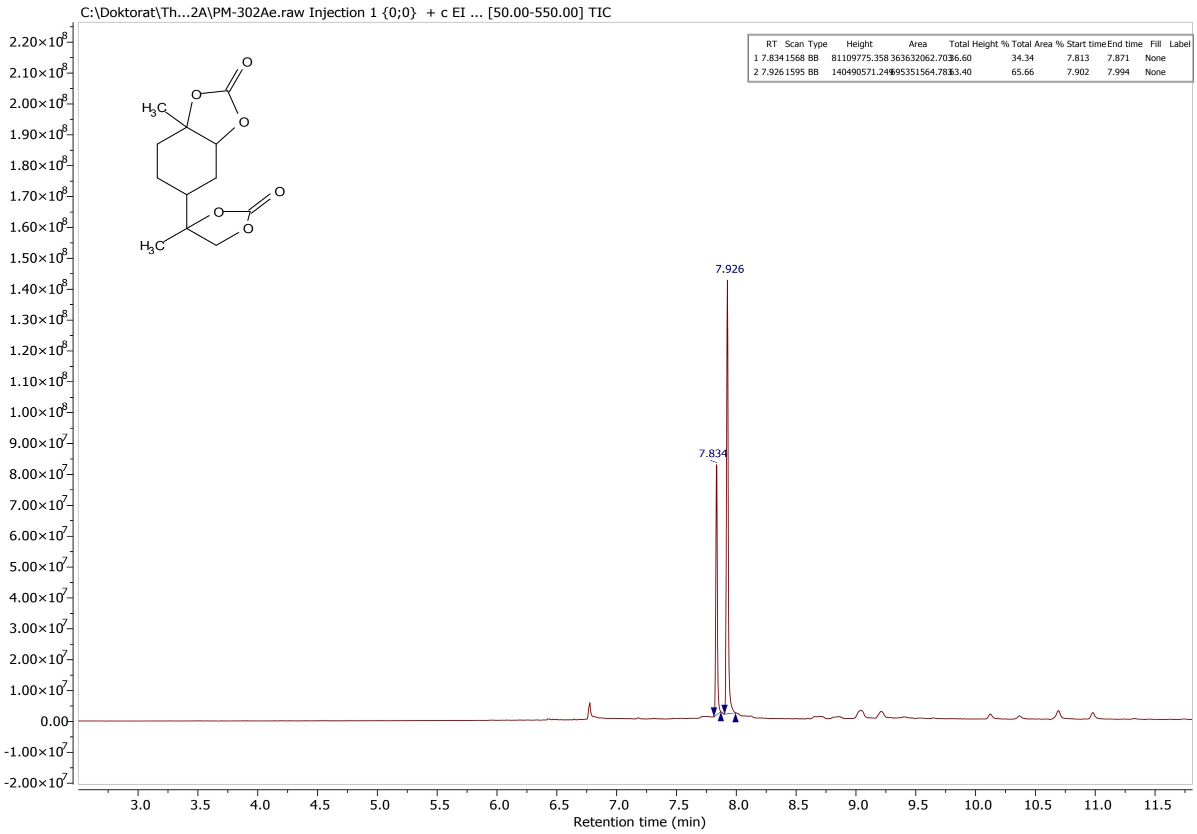

Figure S25: Gas chromatogram of the diastereomeric mixture of biscarbonate 8c.

6 List of Abbreviations

$[C_2mim]Br$	1-ethyl-3-methylimidazolium bromide
$[C_2mim]Cl$	1-ethyl-3-methylimidazolium chloride
$[C_2mim]I$	1-ethyl-3-methylimidazolium iodide
approx.	approximately
aqu.	aqueous
BC	(isomer of) biscarbonate **8c**
BET	Brunauer-Emmett-Teller
BJH	Barret-Joyner-Halenda
br	broad (NMR)
cat.	catalytically / catalyst / catalysis
d	doublet (NMR)
DCM	dichloromethane
dd	doublet of doublets (NMR)
dt	doublet of triplets (NMR)
EC	(isomer of) epoxycarbonate **8b**
equiv.	equivalent
EtOAc	ethyl acetate
GC/MS	gas chromatography - mass spectrometry hyphenation
J	coupling constant (NMR)
LC	limonene carbonate
LO	limonene oxide
LP	light petroleum (boiling point 40 - 60 °C)
m	multiplet (NMR)
m/z	ratio of mass to charge (GC/MS)
M^+	molecular ion (GC/MS)
Me	methyl
NMR	nuclear magnetic resonance
ppm	parts per million
R_f	retention factor (TLC)
rt	room temperature
s	singlet (NMR)
satd.	saturated
$scCO_2$	supercritical CO_2
SILP	supported ionic liquid phase
TBAB	tetrabutylammonium bromide
TBAC	tetrabutylammonium chloride
TBAI	tetrabutylammonium iodide
TGA	thermogravimetric analysis
TLC	thin layer chromatography
UV	ultraviolet
UV-Vis	ultraviolet-visible
wt%	weight percent
δ	chemical shift (NMR)

References

1. Bresien, J.; Ellinger, S.; Harloff, J.; Schulz, A.; Sievert, K.; Stoffers, A.; Täschler, C.; Villinger, A.; Zur Täschler, C., Tetracyanido(difluorido)phosphates M+[PF2(CN)4]−. **2015,** *54* (15), 4474-4477.

2. Shen, M.-L.; Xu, L.; Liu, B.; Jiao, H.; Kwon, Y.-U., A thermosensitive fluorescent Eu-based metal–organic framework and its polyether sulfone composite film as a thermal sensor. *Dalton Transactions* **2018,** *47* (25), 8330-8336.

3. Lin, S.-T.; Ding, M.-F.; Chang, C.-W.; Lue, S.-S., Nuclear magnetic resonance spectroscopic study on ionic liquids of 1-alkyl-3-methylimidazolium salts. *Tetrahedron* **2004,** *60* (42), 9441-9446.

4. Bartlett, P. N.; Cummings, C. Y.; Levason, W.; Pugh, D.; Reid, G., Halometallate Complexes of Germanium(II) and (IV): Probing the Role of Cation, Oxidation State and Halide on the Structural and Electrochemical Properties. **2014,** *20* (17), 5019-5027.

5. Morikawa, H.; Minamoto, M.; Gorou, Y.; Yamaguchi, J.; Morinaga, H.; Motokucho, S., Two Diastereomers of d-Limonene-Derived Cyclic Carbonates from d-Limonene Oxide and Carbon Dioxide with a Tetrabutylammonium Chloride Catalyst. *B Chem Soc Jpn* **2018,** *91* (1), 92-94.
